# Optimizing Viral Discovery in Bats

**DOI:** 10.1371/journal.pone.0149237

**Published:** 2016-02-11

**Authors:** Cristin C. W. Young, Kevin J. Olival

**Affiliations:** 1 University of California Davis, Davis, CA, United States of America; 2 EcoHealth Alliance, New York, NY, United States of America; University of Pretoria, SOUTH AFRICA

## Abstract

Viral discovery studies in bats have increased dramatically over the past decade, yet a rigorous synthesis of the published data is lacking. We extract and analyze data from 93 studies published between 2007–2013 to examine factors that increase success of viral discovery in bats, and specific trends and patterns of infection across host taxa and viral families. Over the study period, 248 novel viruses from 24 viral families have been described. Using generalized linear models, at a study level we show the number of host species and viral families tested best explained number of viruses detected. We demonstrate that prevalence varies significantly across viral family, specimen type, and host taxonomy, and calculate mean PCR prevalence by viral family and specimen type across all studies. Using a logistic model, we additionally identify factors most likely to increase viral detection at an individual level for the entire dataset and by viral families with sufficient sample sizes. Our analysis highlights major taxonomic gaps in recent bat viral discovery efforts and identifies ways to improve future viral pathogen detection through the design of more efficient and targeted sample collection and screening approaches.

## Introduction

Zoonotic disease emergence is driven by a complex web of factors, including human behavior, modifications of natural habitats, changes in agricultural practices, and the underlying pathogen diversity found in animal populations [1]. With increasing environmental disruption and population growth, humans come into contact with bats and other wildlife at an increasing rate, leading to exposure to novel pathogens and disease emergence.

While emerging infectious diseases may spillover from various wildlife species, bats (Order Chiroptera) have been found to be a primary reservoir for numerous recent zoonoses of global concern, such as Ebola, Marburg, Nipah, and Middle Eastern Respiratory Syndrome (MERS-) and Severe Acute Respiratory Syndrome (SARS)-like coronaviruses [2–7]. Second only to rodents in numbers of living genera and species, bats comprise one of the most diverse and ecologically important groups of mammals, with ~1,200 species, accounting for almost a quarter of mammal diversity worldwide [8]. Additionally, studies have found that the life history traits of bats compared to other mammals may make then unique and exceptional hosts for viruses [9, 10]. This possible “uniqueness” of bats to harbor viral pathogens has led to an increased interest in understanding viral diversity and viral richness in bats [9–14].

Beyond the issue of whether bats are “special” in their ability to be reservoirs for zoonotic viruses, there has also been a dramatic increase in the number of general bat viral discovery studies published in the last decade. However, despite the importance of discovering and characterizing novel bat pathogens, at an individual study level most of these efforts have been opportunistic or *ad hoc* in the taxonomic groups and viruses examined. To date, there has been no concerted effort to collate and analyze the methods and findings from these disparate studies using a quantitative approach. Here, we synthesize and analyze patterns of viral discovery in bats from recently published data with the aim of creating a more systematic and efficient approach to identifying novel pathogens. We analyze data from 93 peer-reviewed papers from 2007–2013 using generalized linear mixed models (GLMM) and other approaches to assess the ‘success’ of viral discovery, as well as the probability of detecting a positive sample at an individual level. We identify the study-level variables and methodologies most important for efficient viral discovery, such as specimen type, number of bats sampled, assays used, and bat and viral taxonomy, and explore possible routes of viral shedding and prevalence levels by viral family. We explore several overarching questions, including: what are the overall trends in bat viral discovery over the time period; how many novel viruses were detected over the study period; is viral discovery biased by host or viral taxonomy; what specimen types are most likely to yield specific viruses; and does lethal vs. non-lethal sampling affect the probability of viral detection or the composition of the viral communities discovered?

## Materials and Methods

### Data Sources

Studies were selected using PubMed and Web of Science searches using keywords outlined in **S1 Table** and **S1 Fig** in the **Supporting Information**. Additional references not found in the above searches were added from recent reviews and through references cited in these reviews [9, 15]. A total of 93 primary studies were included in this analysis using our selection criteria. We assembled a database of study level variables for each of the 93 studies that included descriptive statistics, including species of bats collected, viruses found, specimens taken, and viral detection methods used (**S1 and S2 Datasets**). We compiled a separate database with individual data points, where each record in our database (2,565 rows) represented a unique interaction between a virus, host species, sample type, and assay used (**S2 Dataset**). This data structure allowed us to test the probability of detecting a given virus or viral family by sample type, host taxonomy, and other study-level traits.

### Study Inclusion and Exclusion Criteria

We included papers published from January 2007 through June 2013. Papers needed to have at least the following information available in order to be included in the analysis: host identification to at least the species level, sample types tested, virus detection methods used, and viral identification of positive results at least to the family level. Reviews, editorials, and other articles related to bats and viruses but which did not present primary data on viruses in bats were not included. Next generation sequencing (NGS) studies were excluded in all but one instance due to lack of necessary information being reported, including number of host species assessed, and by definition, the number of viral families screened. NGS studies were retained when they followed up with specific PCR assays for given viral families. Furthermore, experimental infection studies were excluded, as were book chapters and articles in languages other than English.

### Data Extraction and Collection

As available, the following variables were extracted from each study or by contacting the corresponding author: year published, country of corresponding author, year(s) sampled, source of bats, number of bat species in the study, number of bats total and number sacrificed, number of specimens tested and number positive, number of novel and total viruses found, number of viral families for which specimens were tested, virus taxonomy (order, family, genus, and species where available), host taxonomy (family, genus, and species where available), number of individual bats per species, sample type, detection method, subsequent tests on a sample, whether the virus was isolated, and whether gene sequencing was undertaken. The source of bats was defined by active surveillance (wild-caught) and passive surveillance (moribund/dead bats, passive surveillance programs, wildlife markets, zoos, rehabilitation centers). Data for bats not identified to species (e.g. to Genus only) were excluded in this analysis. Species were assigned to IUCN categories using the IUCN RedList to one of four categories: Data Deficient, Least Concern, Near Threatened, and Vulnerable [16].

Where attempts to contact corresponding authors were unsuccessful, several necessary assumptions were made throughout the data collation and synthesis, as follows. In three instances, the number of individual bats that tested positive for a virus was not reported, and multiple specimens were positive in multiple species [17–19]. In these cases, we assumed an equal distribution of positive individuals across species. When the total number of bats was not explicitly stated but organ tissue specimens were taken, the total number of bats was assumed to be the total number of specimens [20–24]. When it was not explicitly stated whether the bats captured were lethally sampled, contextual clues were used to assume whether they were or were not sacrificed (e.g., if organ tissue was collected, it was assumed the animals were sacrificed unless explicitly stated that organ biopsies were non-lethal). To address the possibility of sharing of samples among research groups, comparisons were made of authors lists as well as descriptions of the data, including year(s) collected, location collected, and number of specimens collected. Where stated, it was noted whether samples were from archival tissues. Studies that used the same specimens or subsets of specimens were identified and removed. Finally, bats that were found already dead or dying were categorized as non-lethal sampling. This occurred in fifteen studies, with the majority of cases being samples taken from bats that had been found and taken to rehabilitation centers and subsequently died.

### Viral Taxonomy and Novel Virus Designation

Viral taxonomy for previously recognized viruses was synonymized using International Committee on Taxonomy of Viruses (ICTV) v9 [25]. Given the challenge of finding agreement for a taxonomic definition of “novel” viruses, particularly in a meta-analysis of various published studies that use different (non-homologous) genetic makers, we deferred to the authors of each peer-reviewed paper in classifying and defining the number of novel viruses found in each study based on the phylogenetic analysis at the time of publishing. We took a conservative approach in tabulating numbers of viruses, and if peer-reviewed studies did not present sufficient phylogenetic support to clearly differentiate viruses as unique species, we assumed that all strains found represented one viral species.

### Statistical Analyses

We used generalized linear mixed models (GLMM) to fit two separate response variables, the number of novel and total viruses found per study, to first evaluate significant predictors in viral discovery by study design. A negative binomial regression was the best fit for our count data. After testing for collinearity among the response variables in the study-level data, the total number of individual bats in a study was excluded from the model due to its covariance with the number of bat species (r = 0.70, p<0.0001) and the number of specimens tested (r = 0.92, p<0.0001). Explanatory variables included in the full study-level model were: number of species tested, proportion of bats sacrificed, number of total specimens in the study, and number of viral families tested in the study (**Table 1**). Potential confounders such as seasonality were not addressed in our models, as our analysis was limited to raw data from published studies, and most studies did not include the date of collection of individual specimens in their analyses. Backward stepwise methods for variable selection were used along with ranking by Akaike information criterion (AIC) values to identify the best models [26]. We checked model-fitting assumptions using goodness of fit tests including: likelihood ratio χ^2^ tests to assess whether the restricted model fit the data as well as the full model; examination of residual versus fitted value and Q-Q plots; and assessing estimates and confidence intervals for odds ratios for each model (data not shown).

**Table 1:** Variables included in study-level and all data models.

Variable Abbreviation	Variable Description
N_spp	Number of species tested
Prop_Sacrificed	Proportion of bats lethally (vs. non-lethally) sampled per study
N_Specimens	Number of total specimens in the study
ViralFamiliesTest	Number of viral families tested in the study
SampleCat	Specimen type (blood, feces, tissue, urine, saliva, other)
DetMethCat	Detection method (molecular, serology, histopathology, other)
SacrificedNum	Whether the specimen was sacrificed (yes/no)
Virus.Family	Viral taxonomy (Family)
HostFamily	Host taxonomy (Family)
N_Sample_Tested	Number of total specimens tested

We used next used a binomial GLMM (link = logit) to assess the probability of detecting a positive specimen given variables specific to each specimen type, detection method, and study design. Models were fit for the entire dataset, as well as for subsets of data for molecular assays only, serology only, and by each individual viral family. We chose to differentiate between serology only and molecular methods only in our analyses, as serology cannot definitively prove the presence of or previous exposure to specific viral pathogens. Model variables included: specimen type (blood, feces, tissue, urine, saliva, other), detection method (molecular, serology, histopathology, other), whether the specimen was sacrificed (yes/no), host taxonomy (Family), viral taxonomy (Family), number of individual bats, and number of total specimens tested (**Table 1**). The only two variables with significant collinearity were number of individual bats and number of specimens tested (r = 0.99, p<0.0001), and these were not included together during model selection. As before, backward stepwise selection algorithm was run to find the best-fit model for each subset of data and models were ranked by AIC.

We explored patterns of prevalence and calculated mean and median prevalence by specimen type at various viral and host taxonomic levels. All molecular data were aggregated to produce boxplots and heat maps. Heat maps were clustered by similarities in viral richness by row and column using the *hclust* ‘complete linkage’ method in R package *pheatmap* version 0.5.1. Other R packages used included: ggplot2, gplots, plyr, DTK, MASS, Hmisc, and RColorBrewer. All analyses were conducted using R software version 3.1.2 [27].

## Results

### Study-level Data: Summary of Discovery Effort and Temporal Trends

**Fig 1:**
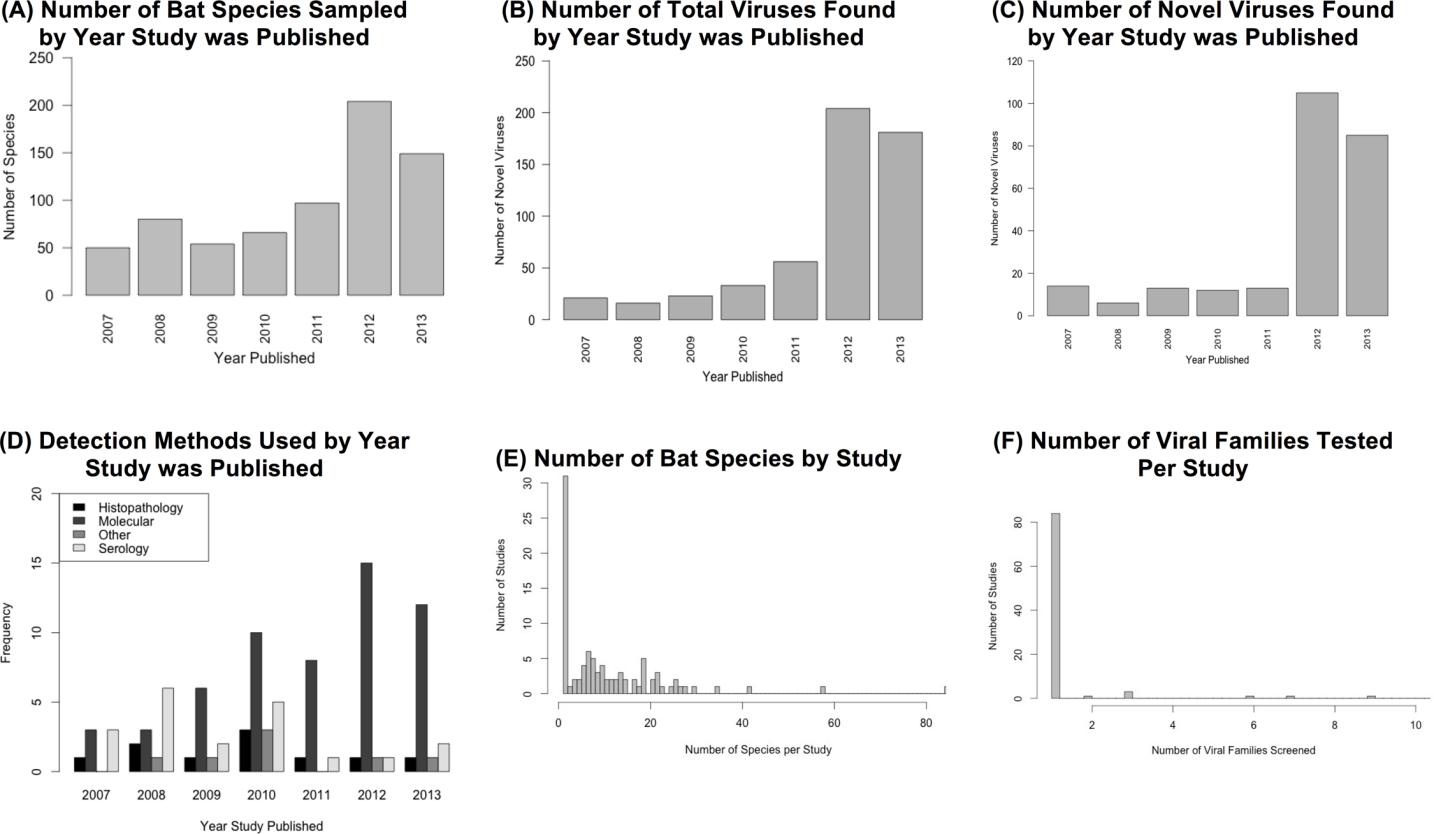
Research and study-level trends. (A) Number of bat species sampled by year study was published; (B) Number of total viruses found by year study was published; (C) Number of novel viruses found by year study was published; (D) Detection methods used by year study was published; (E) Number of bat species by study; (F) Number of viral families tested per study.

A total of 60,416 specimens, from 44,322 bats were collected and tested across all studies from 2007 to mid-2013. Bats from 17 families, 110 genera, and 340 species were sampled for viral discovery across 93 studies (**S2 Table**). Overall, the number of bat species sampled increased during this period (**Fig 1A**), as did the number of novel and total viruses found per year (**Fig 1B and 1C**). A total of 1,891/19,237 (9.83%) specimens were positive by serological assays, and 3,452/155,231 (2.22%) were positive by PCR assays. Viruses from 24 viral families were identified and 248 putative novel viruses were described over the time period. Detection methods have changed, with a decrease in serological assays such as ELISA, and an increase in the use of molecular methods, primarily PCR (**Fig 1D**). There was a wide variance in the number of bat species examined and viral families tested per study (**Fig 1E and 1F**), with most studies examining a single host species and single viral family.

### Study-Level Data: Variables Increasing Viral Discovery ‘Success’

We identified variables predictive of viral discovery ‘success’, as measured by the number of total and novel viruses found at a study level. The best-fit GLMMs (negative binomial regression), for predicting how many novel and total viruses would be found in a given study included, in order of importance, number of species in the study, number of viral families tested, and proportion sacrificed (for total number of viruses only) (AIC = 243.45 and 331.69, respectively, **Table 2**). Although not statistically significant, the fitted model predicted that the lower the proportion of bats sacrificed, the higher the number of total viruses found (0.63, 95% CI 0.39–1.02, p = 0.0657). Subsetting the data by molecular assays only, for a one-unit increase in the number of viral families tested, the fitted model predicted a 29% increase in the number of novel viruses found (1.29, 95% CI 1.15–1.45). The proportion of bats sacrificed and the number of specimens were not significant in the full model for novel viruses, and therefore not included in the fitted models (**Table 2**).

**Table 2:** Best generalized linear mixed models for study-level data–number of novel and total viruses.

Model	AIC	Null deviance	Null d.f.*	Residual deviance	Residual d.f.
**Number of novel viruses**					
All detection methods					
~N_spp + Prop_Sacrificed + N_Specimens + ViralFamiliesTest	247.22	105.83	70	62.83	66
~N_spp + Prop_Sacrificed + ViralFamiliesTest	245.25	105.94	70	62.91	67
~N_spp + ViralFamiliesTest	243.45	105.45	70	62.87	68
Molecular only					
~N_spp + Prop_Sacrificed + N_Specimens + ViralFamiliesTest	223.79	86.51	55	53.44	51
~N_spp + Prop_Sacrificed + ViralFamiliesTest	221.81	86.34	55	53.38	52
~N_spp + ViralFamiliesTest	219.85	86.21	55	53.35	53
**Total number of viruses**					
All detection methods					
~N_spp + Prop_Sacrificed + N_Specimens + ViralFamiliesTest	333.61	195.09	70	57.29	66
~N_spp + Prop_Sacrificed + ViralFamiliesTest	331.69	194.50	70	57.20	67
Molecular only					
~N_spp + Prop_Sacrificed + N_Specimens + ViralFamiliesTest	286.50	145.84	55	48.65	51
~N_spp + Prop_Sacrificed + ViralFamiliesTest	284.53	145.59	55	48.59	52
~N_spp + ViralFamiliesTest	284.20	141.39	55	48.88	53

*d.f. = degrees of freedom.

### Individual-Level Data: Variable Increasing Probability of Detecting Positive Specimen

The best-fit GLMM (logistic regression) for predicting whether a given specimen would be positive or negative included: specimen type, detection method, viral family, and number of specimens tested (AIC = 2015.27, **Table 3**).

**Table 3:** Best generalized linear mixed models for all data–probability of detection.

Model*		AIC	Null deviance	Null d.f.†	Residual deviance	Residual d.f.
**All data**						
Full model	~SampleCat+DetMethCat+SacrificedNum+Virus.Family+ HostFamily+N_Sample_Tested	2017.44	2321.2	1911	1923.4	1865
Stepwise model	~SampleCat+DetMethCat+SacrificedNum+Virus.Family+ N_Sample_Tested	2015.67	2321.2	1911	1951.7	1880
Fitted model	~SampleCat+DetMethCat+Virus.Family+N_Sample_Tested	2015.27	2321.2	1911	1953.3	1881
**All data, molecular only**						
Full model	~SampleCat+SacrificedNum+Virus.Family+HostFamily+ N_Sample_Tested	1608.44	1846.0	1570	1522.4	1528
Stepwise model	~SampleCat+SacrificedNum+Virus.Family+N_Sample_Tested	1597.85	1846.0	1570	1539.9	1542
Fitted model	~SampleCat+Virus.Family+N_Sample_Tested	1595.94	1846.0	1570	1539.9	1543

*Response variable was whether a sample was positive for a given sample.

†d.f. = degrees of freedom.

**Fig 2:**
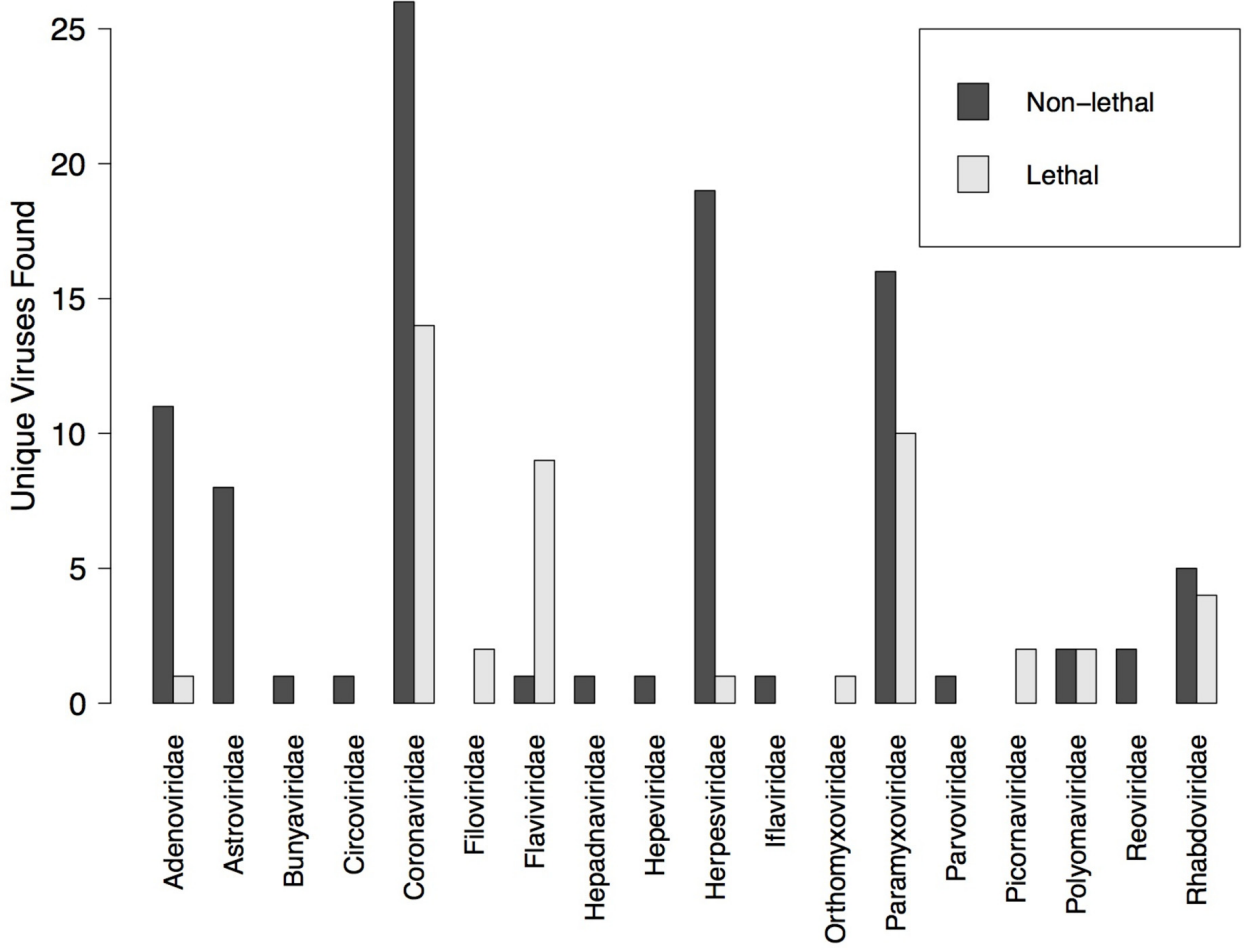
Number of novel viruses found in lethal versus non-lethal studies by viral family.

We found no significant differences in viral detection, when comparing bats that were non-lethally versus lethally sampled. This variable was never significant in our GLMM models (**Table 3**), and even though the number of studies that used lethal vs. non-lethal sampling was roughly equal (49 versus 39), an overall greater number of novel and total viruses were detected across all non-lethal studies (**Fig 2**). With few exceptions (i.e., *Filo*-, *Flavi*-, *Orthomyxo*-, and *Picornaviridae*), non-lethal studies found a greater number of unique viruses in each viral family than studies that used lethal sampling, and many viral families were only detected in non-lethal studies (**Fig 2**). We found that four species of bats sacrificed in viral discovery efforts were listed as vulnerable under the IUCN Red List (*Rousettus obliviosus*, *Taphozous hildegardeae*, *Mormopterus acetabulosus*, and *Myotis capaccinii*), and ten others were listed as near threatened [16].

### Virus and Host Family-Specific Analysis: Probability of Positive Detection

To further explore the probability of detecting a positive specimen for different viral families, data was subsetted by viral family, and separate GLMM models were run. Viral families with enough molecular data to subset included: *Adenoviridae*, *Astroviridae*, *Coronaviridae*, *Flaviridae*, *Hepeviridae*, *Herpesviridae*, *Paramyxoviridae*, *Polyomaviridae*, and *Rhabdoviridae* (**Table 4**). Specimen type was a significant predictor in most models for molecular data, and whether or not a bat was lethally sampled was significant only for *Coronaviridae*, *Herpesviridae*, and *Rhabdoviridae* (**Table 4**). Viral families with serological testing included *Coronaviridae*, *Flaviviridae*, *Paramyxoviridae*, and *Rhabdoviridae*. GLMM models using only serological data all had small sample sizes, and with the exception of *Paramyxoviridae* and *Rhabdoviridae*, the only significant variable in the fitted models was number of specimens tested (**S3 Table and Table 4**).

**Table 4:** Fitted generalized linear mixed models for data subsetted by viral family and viral detection method.

**Subset of data**	Model variables	AIC	Null d.f.*	Residual deviance	Residual d.f.
***Adenoviridae***					
Molecular only	~SampleCat + ViralFamiliesTest + N_Sample_Tested	68.47	66	54.47	60
***Astroviridae***					
Molecular only	~SampleCat + HostFamily	65.58	56	47.58	48
***Coronaviridae***					
Serology only	~N_Sample_Tested	52.99	41	48.99	40
Molecular only	~SampleCat + SacrificedNum + HostFamily + ViralFamiliesTest + N_Sample_Tested	539.83	586	501.83	568
***Flaviviridae***					
Serology only	~N_Sample_Tested	31.92	23	27.92	22
Molecular only	~SampleCat + ViralFamiliesTest + N_Sample_Tested	148.14	148	134.14	142
***Hepeviridae***					
Molecular only	~Null (Intercept Only)	43.40	117	41.40	117
***Herpesviridae***					
Molecular only	~ SacrificedNum + HostFamily + N_Sample_Tested	46.62	100	34.62	95
***Paramyxoviridae***					
Serology only	~ SacrificedNum + ViralFamiliesTest + N_Sample_Tested	80.94	71	72.94	68
Molecular only	~SampleCat + ViralFamiliesTest + N_Sample_Tested	142.45	131	128.45	125
***Polyomaviridae***					
Molecular only	~SampleCat + ViralFamiliesTest + N_Sample_Tested	105.66	102	91.662	96
***Rhabdoviridae***					
Serology only	~HostFamily + N_Sample_Tested	82.62	96	60.62	86
Molecular only	~SampleCat + SacrificedNum + ViralFamiliesTest + N_Sample_Tested	59.002	70	47.002	65

*d.f. = degrees of freedom.

**Fig 3:**
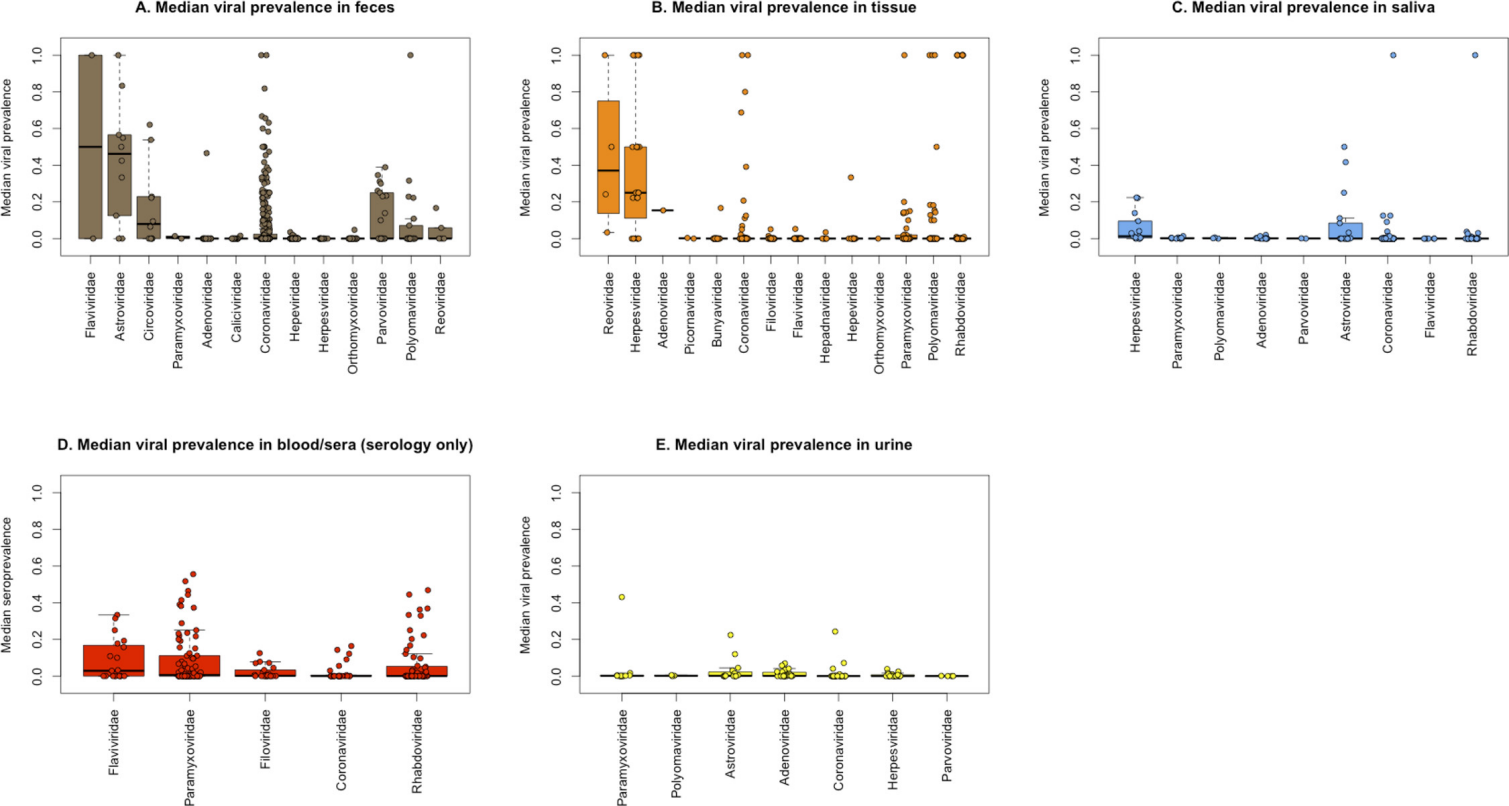
Prevalence of positive specimens by viral family for each specimen type. Boxplots show primary tests only; subsequent tests were not used. (A) Median viral prevalence in feces; (B) Median viral prevalence in tissue; (C) Median viral prevalence in saliva; (D) Median viral prevalence in blood/sera (serology only); (E) Median viral prevalence in urine.

**Fig 4:**
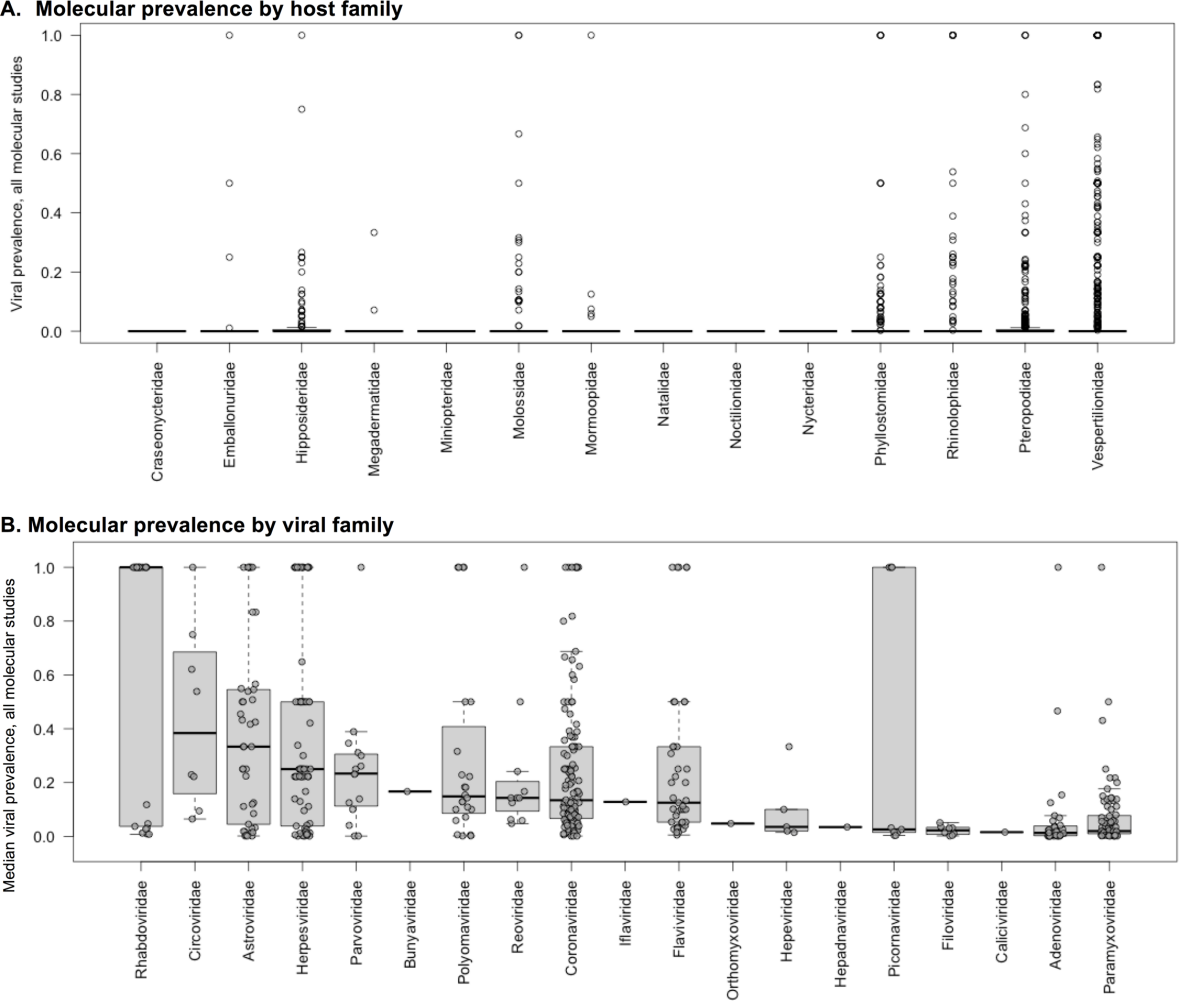
Viral prevalence by host and viral family for all molecular studies. (A) Molecular prevalence by host family; (B) Molecular prevalence by viral family.

Our GLMM results suggest that viral prevalence varied significantly by virus and host families, and by specimen type (**Figs 3 and 4A**). We found a strong sampling bias across both viral and host family, with only a small number of the 24 viral families examined regularly using molecular methods, including *Coronaviridae*, *Paramyxoviridae*, *Astroviridae*, *Circoviridae*, and *Rhabdoviridae*. For viral families with >10 data points, *Astroviridae* and *Circoviridae* had the highest median positive sample prevalence of 33.3% (+/- 5.36%) and 38.3% (+/- 11%), respectively, with other viral families having median prevalences ranging from 1.6–25% (**Fig 4B**). Several data points (unique combinations of host species, specimen type, and assay used for each viral family–see Methods) show 100% prevalence. This upward bias is present across viral families, and is likely due to the inclusion of secondary or nested PCR assays often used to validate findings on subsets of samples found positive in initial screening (**Fig 4B**).

Only six viral families were assayed using serology: *Coronaviridae* (n = 958 specimens), *Filoviridae* (n = 6,882), *Flaviviridae* (n = 616), *Paramyxoviridae* (n = 5,473), *Rhabdoviridae* (n = 5,259); and *Hepeviridae* (n = 49) (**Table 5**). *Coronaviridae* had the highest mean prevalence of positive specimens, while no serological specimens tested positive for *Hepeviridae* (**Table 5**). When evaluating which viral families were found in which specimen types, *Coronaviridae* was the only viral family sampled across all specimen types (**Table 5**). The majority of positive *Coronaviridae* specimens were found in feces through molecular detection methods (PCR), which was validated in our viral family-specific GLMM models (**S3 Table**). Viruses in the family *Flaviviridae* were the only group found equally in blood specimens using both molecular and serological detection methods (n = 88 and n = 73, respectively), with mean prevalences using both detection methods being relatively equal (8.64% and 10.10%, respectively **Table 5**).

**Table 5:** Mean prevalences of specimens tested by detection method stratified by specimen type and viral family.

Viral family	Serological*	Molecular					
	Blood	Blood	Feces	Other	Saliva	Tissue	Urine
*Adenoviridae*	-	-	3.11% (3.11)	100% (-)	0.36%(0.16)	13.82% (9.75)	1.23% (0.36)
*Astroviridae*	-	-	45.13%(8.78)	100% (-)	9.83%(3.94)	4.48% (4.12)	2.84% (1.51)
*Bunyaviridae*	-	-	-	-	-	0.43% (0.43)	-
*Caliciviridae*	-	-	0.11% (0.11)	-	-	-	-
*Circoviridae*	-	-	17.69% (7.28)	100% (-)	-	12.50% (12.50)	-
*Coronaviridae*	22.69% (5.42)	1.04% (1.04)	5.99% (0.78)	33.33%(16.67)	1.83% (1.32)	5.02% (1.95)	1.31% (0.90)
*Filoviridae*	2.59% (0.79)	-	-	-	0.00%(-)	0.57% (0.23)	-
*Flaviviridae*	8.64% (2.20)	9.51% (2.29)	50% (50.00)	100% (-)	2.86%(2.86)	1.52% (0.97)	0.00% (-)
*Hepadnaviridae*	-	-	-	-	-	0.57% (0.57)	-
*Hepeviridae*	0.00% (-)	0.26% (0.26)	0.10% (0.06)	-	-	2.56% (2.56)	-
*Herpesviridae*	-	-	0.00% (-)	100% (-)	6.03%(2.31)	40.38%(4.58)	0.48% (0.18)
*Iflaviridae*	-	-	-	-	-	2.14% (2.14)	-
*Orthomyxoviridae*	-	-	0.21% (0.21)	-	-	0.00% (-)	-
*Papillomaviridae*	-	-	-	100% (-)	-	-	-
*Paramyxoviridae*	10.49% (1.58)	0.22% (0.15)	0.47% (0.47)	-	0.66%(0.48)	6.13% (2.35)	1.76% (1.48)
*Parvoviridae*	-	-	12.19%(3.14)	100% (-)	0.07%(0.07)	1.38% (1.07)	0.027% (0.027)
*Picornaviridae*	-	-	-	100% (-)	-	0.48% (0.21)	-
*Polyomaviridae*	-	-	9.27% (4.97)	0.45% (0.45)	0.26%(0.17)	8.00% (2.84)	0.22% (0.15)
*Poxviridae*	-	-	-	-	-	16.67% (-)	-
*Reoviridae*	-	-	4.51% (3.25)	-	-	31.26%(10.94)	-
*Retroviridae*	-	-	-	100% (-)	-	-	-
*Rhabdoviridae*	5.88% (1.43)	4.69% (-)	-	-	2.44%(2.17)	22.07%(4.69)	-
*Togaviridae*	-	-	-	-	-	0.00%(-)	-

*Mean (%) and standard error.

**Fig 5:**
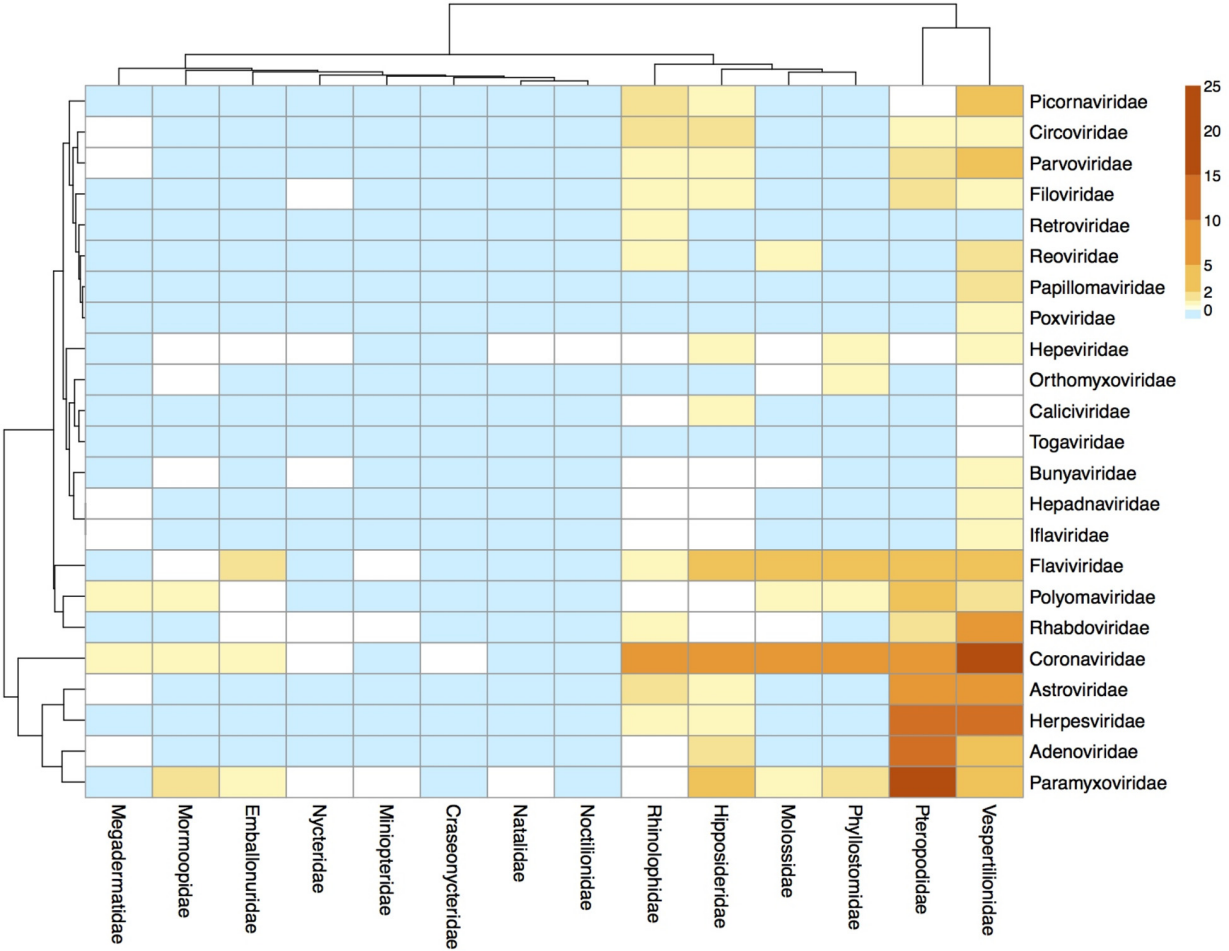
Heat map of viral richness by host and viral families, clustered by host and viral families.

To further explore “surveillance gaps” and viral richness that has been catalogued over the study period, we generated a heat map showing the number of unique viruses found in molecular studies clustered by host and viral family according to similarities in viral richness (**Fig 5**). The greatest number of viruses over the study period were detected in host families that are also the most species rich: *Pteropodidae*, *Vespertilionidae*, *Rhinolophidae*, *Phyllostomidae*, and *Hipposideridae* (**Fig 5**). Among viral families, *Paramyxo*-, *Adeno*-, *Herpes*-, *Astro*-, and *Coronaviridae* had the greatest number of unique viruses detected. Host family-specific heat maps were generated for *Vespertilionidae* and *Pteropodidae*, the two most species-rich and heavily sampled bat families in our dataset (**S2 and S3 Figs**). Particularly rich viral families in both *Vespertilionidae* and *Pteropodidae* included *Adeno-*, *Astro-*, *Corona-*, *Herpes-*, and *Paramyxoviridae*. We observed a large bias in surveillance effort and pathogen discovery success in the dataset, with only a fraction of the potential surveillance space (i.e. host genus/virus family combinations) examined over study period for *Pteropodidae* (14.2% of host genus/viral family dyads tested; 6.3% found positive for at least one virus) and *Vespertilionidae* (21.9% tested; 9.5% positive) (**S2 and S3 Figs**).

## Discussion

In this large, quantitative review of bat viral discovery studies we show significant differences in viral prevalence and discovery success across host and viral taxonomic levels, and use a GLMM approach to identify the specimen types and other variables most likely to yield positive detections for key viral families. These data can be used to streamline future bat viral discovery efforts through better study design, including targeting by specimen type when looking for specific viral pathogens, the adoption of non-invasive field collection methods, identification of taxonomic gaps in discovery efforts, and in focusing laboratory effort to the host species and specimens most likely to result in detection for pathogens of interest.

### Overall Patterns of Viral Richness and Surveillance Gaps

Our quantitative review of viral discovery efforts in bats includes data from over 44,000 individual bats from 340 species for 24 different viral families. We found that the number of bat viruses discovered over time has increased, and ~250 putatively novel viruses were discovered over the study period. However, we identify significant surveillance biases over this period, and highlight these taxonomic gaps as opportunities for future research in the coming decade. While viruses from 24 viral families were identified in 17 bat families across all studies, only a small fraction of surveillance space (~25% of bat taxa—viral family dyads) was examined, and viral richness varied across host taxonomy at a bat family and bat genus level. This later observation is a likely driven by a combination of both differences in host species richness and host abundance and feasibility of capture (e.g. *Vespertilionidae* bats are the second largest mammalian family and geographically the most widespread bat family [8]), but it may also reflect inherent differences in viral richness among bat species based on life history and ecological traits [9, 28, 29].

However, our models excluded host taxonomy as a significant predictor of whether or not a given specimen would be positive. We further showed that mean viral prevalence did not significantly differ across bat families even though some bat taxa were much more heavily sampled than others and had subsequently greater observed viral richness.

We observed a change in viral detection methods used over time with an increase in PCR-based approaches and a growing adoption of NGS. In the last few years, NGS or metagenomic methods have become more commonplace in the bat viral discovery field. Although these results are valuable to the literature, we excluded all but one of these studies from our current analyses, as we were not able to extract comparable variables from these studies. For example, the output or response variables were not directly comparable (thousands of short sequence reads often grouped by viral family for NGS studies versus definitive positive or negatives for PCR screening and sequencing studies). We believe that closer examination of NGS studies used for bat pathogen discovery warrants a separate analysis; one that we hope the research community will undertake in the next few years.

We also observed a decline in the use of serology and viral isolation. Reporting of virus isolation was sporadic across studies, with only 24 explicitly stating that they had isolated virus from bat specimens. Virus isolation may be less frequently done for several reasons; it can be labor intensive and there are limited laboratories with suitable containment capabilities. However, there are potential limitations of not performing viral isolation. For example, complete phenotypic characterization of a virus cannot be done without isolation, and whether a virus is “novel” cannot be truly known with only viral genomic sequences [30].

Only five viral families were detected using serology (out of six tested), compared to 20 out of 22 viral families using molecular detection techniques. Expanding the development of more sensitive and virus-specific serological assays, ideally with the capacity to multiplex across a large number of bat pathogens would help better inform disease dynamics in natural populations of bats. Existing platforms, e.g. Luminex and LIPS assays, should be expanded to include novel pathogens recently discovered in bats that are of high interest [31, 32]. However, serological testing to detect certain viruses in bats should be interpreted with caution, as there are several potential limitations to serological assays. Most notably, cross-reactivity of antibodies to multiple pathogens can lead to decreased assay specificity and confounding results for closely related viruses [33]. Additionally, there is a lack of standardization for cutoff levels across assays, leading to varied interpretations of results [33].

### Study-Level Models

Our findings from the study-level regression analyses suggest that future viral discovery efforts would be most productive if focused on testing a broader array of bat species and viral families. Notably, both the proportion of bats sacrificed and the number of specimens examined both dropped out of the final fitted model when both the number of total and novel viruses were used as response variables. This suggests that viral discovery efforts will be maximized by including more species of bats, instead of (or in addition to) more specimens of individuals from the same bat species. While not an entirely novel finding, these data suggest that we have not yet saturated the bat viral discovery curve for most species. This was demonstrated by intensive sampling in a single species of bat (*Pteropus giganteus*) which produced the discovery of 55 viruses from seven viral families, and showed that nearly 7,000 specimens are required to fully saturate the viral discovery curve for that species [34]. While Anthony et *al*. collected over 2,000 specimens over four years; less than 20% of the studies in our analysis had sample sizes greater than 1,000. While the intensive approach of longitudinal sampling and collection of thousands of specimens might be ideal for quantifying the total diversity of viruses in a bat species, our findings suggests that for studies without extensive resources, including more species of bats and testing a broader number of viral families alone are efficient ways to discover more viruses.

### Lethal Sampling in Bats

Our analysis found that there were no significant differences between studies that used lethal vs. non-lethal sampling techniques in terms of numbers of novel and total viruses discovered, and that this variable was not part of the best model that predicted probability of virus detection. Our raw data showed that a greater number of viruses were found in studies that did not sacrifice bats, with a few exceptions, including Filoviruses and Orthomyxoviruses. However, this is likely due to bias in surveillance methods to date; for example, most Filovirus studies we examined did not attempt to screen non-lethally collected bat excreta. Recent data from experimental studies show that Marburg virus is shed in the saliva of *Rousettus* sp. bats [35], which points to a non-lethal specimen type that may be productive for Filovirus detection and discovery in the future. Our findings suggest that the use of recently developed protocols for non-lethal sampling for pathogen discovery may be valuable (33, 34), and global efforts that have adopted these methods for wildlife viral discovery may find success in their efforts (35).

While the approach we outline may be of the best value for overall viral discovery success in bats, targeted and sometimes lethal sampling approaches may be justified and more effective depending on the pathogen in question or the research question. For example, European Bat Lyssavirus is most commonly detected in brain tissue especially from symptomatic animals [36, 37], and may be more productive and cost-effective for identifying this rhabdovirus from species and regions of concern than active surveillance using oral swabs. Focused investigations on bats that are moribund or found recently dead may be more likely to yield viral detections than lethal sampling of apparently healthy bats in some instances, as is the case for rabies and other lyssaviruses, where viruses are known to produce clinical symptoms in bats.

In some cases, targeted, lethal sampling may be necessary for both viral detection and for vouchering of host specimens. Lethal sampling is also required in experimental infection studies in bats. These studies themselves are critical for understanding pathogen tissue tropism and routes of excretion in particular host-pathogen models, and can ultimately help inform specimen types to target for field surveillance. However, when studies are focused on virome-wide sampling for pathogen discovery, or sampling rare or endangered species, we argue that non-lethal sampling is best and in line with bat conservation activities and can be a productive approach for pathogen discovery.

### Gaps in Data Reporting and Areas for Future Research

During our data collection, we found inconsistencies in methodologies and reporting that would have improved the power of our analysis, and would have allowed for the inclusion of more studies for comparison. For example, several studies mentioned only the number of bats sampled or the number of specimens taken, not both. Furthermore, specimens were often not broken down by bat species or specimen type and detection method used. We also found that identification of bats to the species, or even genus, level was often incomplete–highlighting the need for virologists to collaborate better with bat taxonomic experts and those with extensive field experience. Several studies we initially examined used the same set of specimens for several publications. This is a potential confounder of which to be aware; however, after closer investigation all but one pair of studies that used duplicate samples examined different viral families, and this study was excluded from our analysis. Finally, we cannot rule out the possibility that collaborators shared samples among their research groups, with the samples’ origin with regard to potential lethal sampling not being clarified or adequately reported in subsequent studies using these samples. Open communication and collaboration among and between research groups with regard to sample origins as well as meticulous reporting will help to resolve this issue in future publications.

We also observed taxonomic bias in surveillance by host family; while 17 host families were sampled across all studies, our analysis found that the majority of specimens tested were from only a few bat families. Research collaborations between wildlife and conservation biologists and virologists, using non-invasive sampling methods, will be needed to fill in gaps for rare and understudied bat taxa and obtain a more complete the picture of bat viral richness.

We show virus-family specific differences in prevalence by specimen type. These findings are of value to help target high-yield specimen types to increase efficiently of detection when a specific pathogen group is targeted and resources are limited. For example, while roost urine collection may be effective for some specific viruses and hosts (e.g. Henipavirus detection in *Pteropus* [38]) and the collection method is non-invasive and relatively inexpensive, our analysis found that urine specimens yielded low mean viral prevalence (<3% overall), despite large sample sizes. The broadest ranges of viruses were tested for in tissue specimens (20/24 viral families), and while we recognize that all tissues types are not likely to yield the same viral communities, due to the structure and availability of individual-level data, we pooled all tissue types when collating the data presented here. More detailed analysis of viral discovery by tissue type may lead to better knowledge of tissue tropism for certain viral families.

Overall, there is a dearth of information for more than half of the 24 viral families as to what specimen type may be most likely to yield viral detection. Future research could focus on testing for more viral families across the various types of excreta or on experimental infections, both of which will improve our understanding of routes of viral transmission and viral shedding in bats. This sampling bias will only be fixed with more targeted efforts in the field and laboratory to address these surveillance gaps. Notably, many of the viral families in our analysis have been targeted because of their public health significance, i.e. the increase in coronavirus research after the emergence of SARS-CoV and MERS-CoV, and thus are over-represented in our dataset relative to other viral groups. The use of novel and unbiased methods such as NGS and VirCapSeq-VERT, aimed at detecting all mammalian viruses, will help elucidate more unbiased patterns of viral patterns between bat species and across global bat populations [39].

We also acknowledge that our analysis of viral prevalence may be biased by uneven geographic sampling, not just by the effect of host taxonomy and specimen type as we show, as there may be particular regions of there world and bat communities that have been over-sampled for particular viral families, e.g. coronaviruses in China [4, 40–42]. Poor reporting of sampling locality information precluded us from testing this explicitly. Lastly, our compiled estimates of mean prevalence by viral family and sample type can be used to design statistically valid field investigations, particularly in planning minimum sample sizes necessary for pathogen detection or to obtain statistically significant estimates of prevalence with adequate power.

We acknowledge a key caveat in our estimate of the number of novel viruses found over the study period. The designation of ‘novel’ for each virus discovered was based on small gene fragments and phylogenetic analysis conducted by individual authors and the peer reviewed system at the time of publication. If two separate studies examine non-homologous gene regions for a given virus, these studies may be ‘double counting’ the existing known number of viruses, and thus leading to overestimates in our analysis of pooled data. As previously described, we were conservative in our estimates of novel viruses and assumed all strains found constituted one viral species unless well-supported phylogenetic data from the peer-reviewed studies suggested otherwise. Nevertheless, more consistent reporting, vouchering, and, ultimately, full genome analysis of viral discoveries may help to alleviate this limitation in the future [30].

### Conclusions

We provide a synopsis and quantitative review of the burgeoning field of bat virology, with implications for how future viral discovery studies in bats are designed, including how specimens are collected and whether or not bats should be sacrificed to obtain specimens. Lethal sampling does not appear to increase success of obtaining a positive viral detection, and future studies may focus on developing improved non-lethal sampling methods, thereby helping to ensure conservation of bat populations. We show clear differences in viral prevalence and detection probability by specimen type and host taxonomy, and identified taxonomic gaps where viruses have not been screened and in those where viruses have been screened but not yet discovered. We hope these data will begin to streamline future viral discovery efforts through more targeted collection of specimens, obtaining statistically significant, adequately powered sample sizes, increased research for currently under-represented bat taxa, and in targeting laboratory assays to the species and specimens most likely to result in pathogen detection and discovery.

## Supporting Information

S1 Dataset Study-level data used for analyses. (CSV)

S2 Dataset Individual-level data used for analyses. (CSV)

S1 Fig Identification of eligible bat virus studies. (DOCX)

S2 Fig Heat map of viral richness for *Vespertilionidae*, clustered by host genus and viral family. (DOCX)

S3 Fig Heat map of viral richness for *Pteropodidae*, clustered by host genus and viral family. (DOCX)

S1 Table Databases/Reviews and filters used to find studies. (DOCX)

S2 Table Studies examined and study-level characteristics. (DOCX)

S3 Table Fitted generalized linear mixed models for data subsetted by viral family and detection method, with coefficients. (DOCX)

## References

[1] Morse SS, Mazet JA, Woolhouse M, Parrish CR, Carroll D, Karesh WB, et al Prediction and prevention of the next pandemic zoonosis. The Lancet. 2012;380(9857):1956–65. 10.1016/S0140-6736(12)61684-5 PMC3712877 23200504

[2] Olival KJ, Hayman DT. Filoviruses in Bats: Current Knowledge and Future Directions. Viruses. 2014;6(4):1759–88. 10.3390/v6041759 24747773 PMC4014719

[3] Memish ZA, Mishra N, Olival KJ, Fagbo SF, Kapoor V, Epstein JH, et al Middle East respiratory syndrome coronavirus in bats, Saudi Arabia. Emerg Infect Dis. 2013;19(11):1819–23. 10.3201/eid1911.131172 24206838 PMC3837665

[4] Ge XY, Li JL, Yang XL, Chmura AA, Zhu G, Epstein JH, et al Isolation and characterization of a bat SARS-like coronavirus that uses the ACE2 receptor. Nature. 2013;503(7477):535–8. 10.1038/nature12711 . 24172901 PMC5389864

[5] Rahman SA, Hassan SS, Olival KJ, Mohamed M, Chang LY, Hassan L, et al Characterization of Nipah virus from naturally infected Pteropus vampyrus bats, Malaysia. Emerg Infect Dis. 2010;16(12):1990–3. 10.3201/eid1612.091790 21122240 PMC3294568

[6] Wynne JW, Wang LF. Bats and viruses: friend or foe? PLoS Pathog. 2013;9(10):e1003651 10.1371/journal.ppat.1003651 24204253 PMC3814676

[7] Pourrut X, Souris M, Towner JS, Rollin PE, Nichol ST, Gonzalez J-P, et al Large serological survey showing cocirculation of Ebola and Marburg viruses in Gabonese bat populations, and a high seroprevalence of both viruses in Rousettus aegyptiacus. BMC infectious diseases. 2009;9:159 10.1186/1471-2334-9-159 . 19785757 PMC2761397

[8] Wilson DE, Reeder DM. Mammal species of the world: a taxonomic and geographic reference 3rd ed. Baltimore: Johns Hopkins University Press; 2005.

[9] Luis AD, Hayman DTS, O'Shea TJ, Cryan PM, Gilbert AT, Pulliam JRC, et al A comparison of bats and rodents as reservoirs of zoonotic viruses: are bats special? Proceedings Biological sciences / The Royal Society. 2013;280:20122753 10.1098/rspb.2012.2753 . 23378666 PMC3574368

[10] O'Shea TJ, Cryan PM, Cunningham AA, Fooks AR, Hayman DT, Luis AD, et al Bat flight and zoonotic viruses. Emerg Infect Dis. 2014;20(5):741–5. 10.3201/eid2005.130539 24750692 PMC4012789

[11] Olival KJ, Epstein JH, Wang L-F, Field HE, Daszak P. Are bats exceptional viral reservoirs. New directions in conservation medicine: Applied cases of ecological health. 2012:195–212.

[12] Wang LF, Walker PJ, Poon LL. Mass extinctions, biodiversity and mitochondrial function: are bats 'special' as reservoirs for emerging viruses? Curr Opin Virol. 2011;1(6):649–57. 10.1016/j.coviro.2011.10.013 . 22440923 PMC7102786

[13] Luis AD, O'Shea TJ, Hayman DT, Wood JL, Cunningham AA, Gilbert AT, et al Network analysis of host–virus communities in bats and rodents reveals determinants of cross‐species transmission. Ecology letters. 2015;18(11):1153–62. 26299267 10.1111/ele.12491 PMC5014217

[14] Dobson AP. Virology. What links bats to emerging infectious diseases? Science. 2005;310(5748):628–9. 10.1126/science.1120872 . 16254175

[15] Daszak P, Bogich T, Hosseini P, Zambrana-Torrellio C, Olival K, Mazet J, et al Modeling Risk: The Use of Geo-Temporal Models for Focusing Risk Reduction Interventions. EcoHealth. 2011;7:S135–S. .

[16] IUCN Red List of Threatened Species [Internet]. IUCN. 2013. Available from: http://www.iucnredlist.org/.

[17] Sonntag M, Mühldorfer K, Speck S, Wibbelt G, Kurth A. New adenovirus in bats, Germany. Emerging infectious diseases. 2009;15:2052–5. 10.3201/eid1512.090646 . 19961700 PMC3044533

[18] Drexler JF, Corman VM, Muller M, Maganga G, Vallo P, Binger T, et al Bats host major mammalian paramyxoviruses. Nature communications. 2012;3:796 10.1038/ncomms1796 22531181 PMC3343228

[19] Kurth A, Kohl C, Brinkmann A, Ebinger A, Harper JA, Wang L-F, et al Novel paramyxoviruses in free-ranging European bats. PloS one. 2012;7:e38688 10.1371/journal.pone.0038688 22737217 PMC3380927

[20] Kohl C, Lesnik R, Brinkmann A, Ebinger A, Radonic A, Nitsche A, et al Isolation and characterization of three mammalian orthoreoviruses from European bats. PloS one. 2012;7:e43106 10.1371/journal.pone.0043106 22905211 PMC3419194

[21] Kuzmin IV, Mayer A, Niezgoda M, Markotter W, Agwanda B, Breiman RF, et al Shimoni bat virus, a new representative of the Lyssavirus genus. Virus research. 2010;149:197–210. 10.1016/j.virusres.2010.01.018 20138934

[22] Kuzmin IV, Niezgoda M, Franka R, Agwanda B, Markotter W, Breiman RF, et al Marburg virus in fruit bat, Kenya. Emerging infectious diseases. 2010;16:352–4. 10.3201/eid1602.091269 . 20113584 PMC2958024

[23] Lelli D, Moreno A, Lavazza A, Bresaola M, Canelli E, Boniotti M, et al Identification of Mammalian orthoreovirus type 3 in Italian bats. Zoonoses and public health. 2013;60:84–92. 10.1111/zph.12001 22931153

[24] Emerson GL, Nordhausen R, Garner MM, Huckabee JR, Johnson S, Wohrle RD, et al Novel Poxvirus in Big Brown Bats, Northwestern United States—Vol. 19 No. 6—June 2013—Emerging Infectious Disease journal—CDC. Emerg Infect Dis. 2013;19:1002–4. 10.3201/eid1906.121713 23735421 PMC3713833

[25] International Committee on Taxonomy of Viruses., King AMQ. Virus taxonomy: classification and nomenclature of viruses: ninth report of the International Committee on Taxonomy of Viruses London; Waltham, MA: Academic Press; 2012 x, 1327 p. p.

[26] Rodríguez G. Generalized Linear Models Princeton University2013. Available from: http://data.princeton.edu/R/glms.html.

[27] R Development Core Team R. R: A language and environment for statistical computing Vienna, Austria: R Foundation for Statistical Computing; 2013.

[28] Turmelle AS, Olival KJ. Correlates of Viral Richness in Bats (Order Chiroptera). EcoHealth. 2009;6(4):522–39. 10.1007/s10393-009-0263-8 20049506 PMC7088156

[29] Gay N, Olival KJ, Bumrungsri S, Siriaroonrat B, Bourgarel M, Morand S. Parasite and viral species richness of Southeast Asian bats: Fragmentation of area distribution matters. International journal for parasitology Parasites and wildlife. 2014;3(2):161–70. 10.1016/j.ijppaw.2014.06.003 25161915 PMC4142259

[30] Calisher CH, Tesh RB. Two misleading words in reports of virus discovery: little things mean a lot. Archives of virology. 2014;159(8):2189–91. 10.1007/s00705-014-2008-4 . 24532301

[31] Bossart KN, McEachern JA, Hickey AC, Choudhry V, Dimitrov DS, Eaton BT, et al Neutralization assays for differential henipavirus serology using Bio-Plex protein array systems. Journal of virological methods. 2007;142(1–2):29–40. 10.1016/j.jviromet.2007.01.003 . 17292974

[32] Burbelo PD, Ching KH, Klimavicz CM, Iadarola MJ. Antibody profiling by Luciferase Immunoprecipitation Systems (LIPS). Journal of visualized experiments: JoVE. 2009;(32). 10.3791/1549 19812534 PMC3164068

[33] Gilbert AT, Fooks AR, Hayman DT, Horton DL, Muller T, Plowright R, et al Deciphering serology to understand the ecology of infectious diseases in wildlife. EcoHealth. 2013;10(3):298–313. 10.1007/s10393-013-0856-0 . 23918033

[34] Anthony SJ, Epstein JH, Murray KA, Navarrete-Macias I, Zambrana-Torrelio CM, Solovyov A, et al A strategy to estimate unknown viral diversity in mammals. mBio. 2013;4(5):e00598–13. 10.1128/mBio.00598-13 24003179 PMC3760253

[35] Amman BR, Jones ME, Sealy TK, Uebelhoer LS, Schuh AJ, Bird BH, et al Oral shedding of marburg virus in experimentally infected egyptian fruit bats (rousettus aegyptiacus). Journal of wildlife diseases. 2015;51(1):113–24. 10.7589/2014-08-198 . 25375951 PMC5022530

[36] Amengual B, Bourhy H, López-Roig M, Serra-Cobo J. Temporal dynamics of European bat Lyssavirus type 1 and survival of Myotis myotis bats in natural colonies. PloS one. 2007;2:e566 10.1371/journal.pone.0000566 . 17593965 PMC1892799

[37] Banyard AC, Hayman D, Johnson N, McElhinney L, Fooks AR. Bats and lyssaviruses. Advances in virus research. 2011;79:239–89. Epub 2011/05/24. 10.1016/b978-0-12-387040-7.00012-3 . 21601050

[38] Wacharapluesadee S, Boongird K, Wanghongsa S, Ratanasetyuth N, Supavonwong P, Saengsen D, et al A longitudinal study of the prevalence of Nipah virus in Pteropus lylei bats in Thailand: evidence for seasonal preference in disease transmission. Vector borne and zoonotic diseases (Larchmont, NY). 2010;10:183–90. 10.1089/vbz.2008.0105 . 19402762

[39] Briese T, Kapoor A, Mishra N, Jain K, Kumar A, Jabado OJ, et al Virome Capture Sequencing Enables Sensitive Viral Diagnosis and Comprehensive Virome Analysis. mBio. 2015;6(5):e01491–15. 10.1128/mBio.01491-15 26396248 PMC4611031

[40] Lau SK, Li KS, Tsang AK, Shek CT, Wang M, Choi GK, et al Recent transmission of a novel alphacoronavirus, bat coronavirus HKU10, from Leschenault's rousettes to pomona leaf-nosed bats: first evidence of interspecies transmission of coronavirus between bats of different suborders. Journal of virology. 2012;86(21):11906–18. Epub 2012/08/31. 10.1128/jvi.01305-12 22933277 PMC3486284

[41] Woo PC, Lau SK, Lam CS, Lau CC, Tsang AK, Lau JH, et al Discovery of seven novel Mammalian and avian coronaviruses in the genus deltacoronavirus supports bat coronaviruses as the gene source of alphacoronavirus and betacoronavirus and avian coronaviruses as the gene source of gammacoronavirus and deltacoronavirus. Journal of virology. 2012;86(7):3995–4008. Epub 2012/01/27. 10.1128/jvi.06540-11 22278237 PMC3302495

[42] Wu Z, Ren X, Yang L, Hu Y, Yang J, He G, et al Virome analysis for identification of novel mammalian viruses in bat species from Chinese provinces. Journal of virology. 2012;86(20):10999–1012. Epub 2012/08/03. 10.1128/jvi.01394-12 22855479 PMC3457178

