# Supplementary material for: Optimizing Viral Discovery in Bats
Source: PLoS One. 2016 Feb 11;11(2):e0149237. doi: 10.1371/journal.pone.0149237 (PMC4750870; doi:10.1371/journal.pone.0149237)
Supplement: S1 Fig — (DOCX) [file pone.0149237.s003.docx]

**Figure S1. Identification of eligible bat virus studies**

**PubMed** search: “bat AND (virus OR viral)”, 2007-2013

**459** results

**Web of Science** search: “bat AND (virus OR viral) AND (novel OR emerging OR new)” refined by doc type (article), 2007-2013

**846** results

Results refined based on duplicate results, titles and abstracts

**102** results

Luis et al. review:

2007-current

**131** results

Existing database (data not published): 2007-current

**59** results

**162** results provisionally included

**93** results included

**68** results excluded:

**24** - original article not available

**19** - bat/virus data not available

**4** - in a language other than English

**22** - other

**76** results excluded based on title

**34** duplicate results

**20** duplicate results
