# Supplementary material for: Optimizing Viral Discovery in Bats
Source: PLoS One. 2016 Feb 11;11(2):e0149237. doi: 10.1371/journal.pone.0149237 (PMC4750870; doi:10.1371/journal.pone.0149237)
Supplement: S2 Fig — (DOCX) [file pone.0149237.s004.docx]

F**igure S2. Heat map of viral richness for *Vespertilionidae*, clustered by host genus and viral family**

**
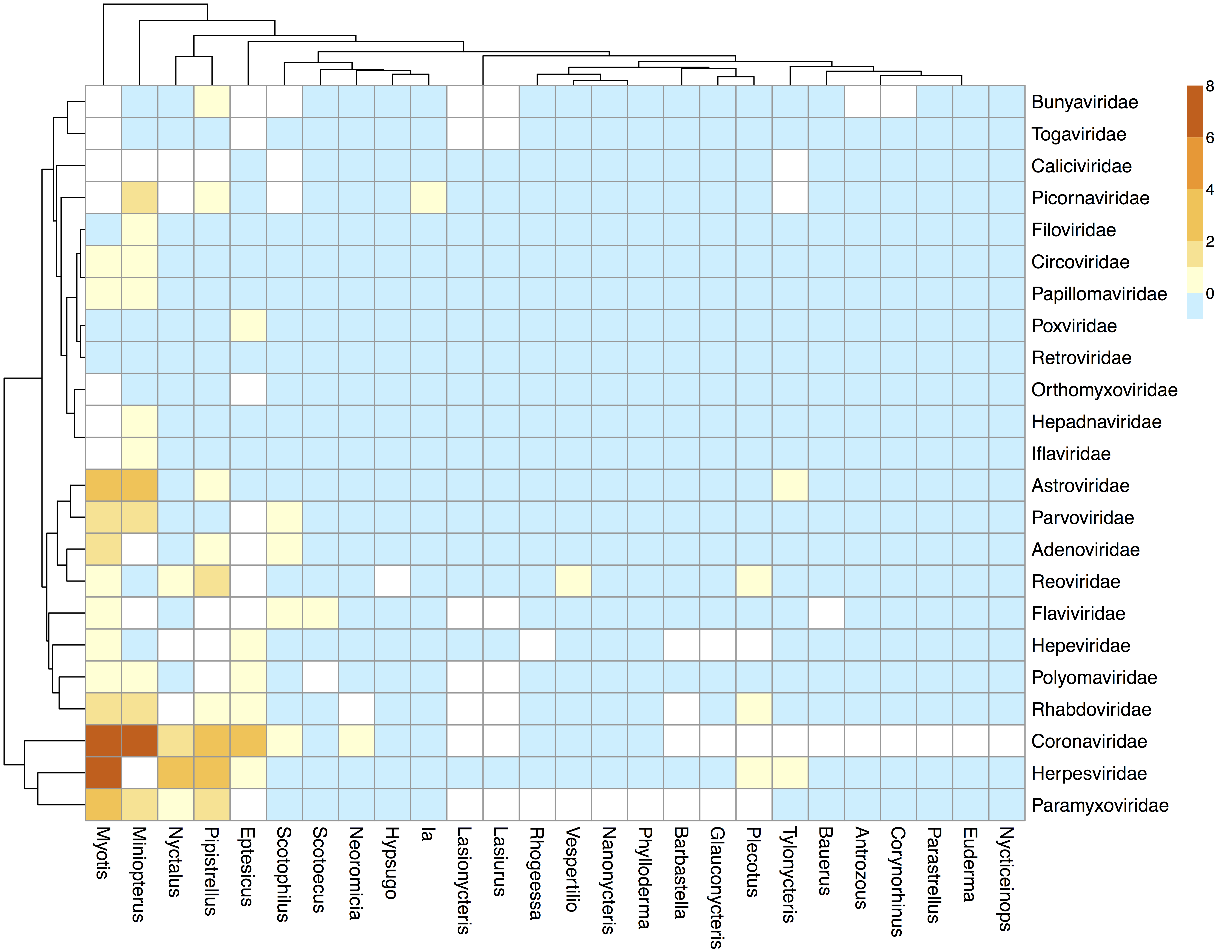
**
