# Supplementary figures and images for: Optimizing Viral Discovery in Bats
Source: PLoS One. 2016 Feb 11;11(2):e0149237. doi: 10.1371/journal.pone.0149237 (PMC4750870; doi:10.1371/journal.pone.0149237)

**Figure S3. Heat map of viral richness for *Pteropodidae*, clustered by host genus and viral family**


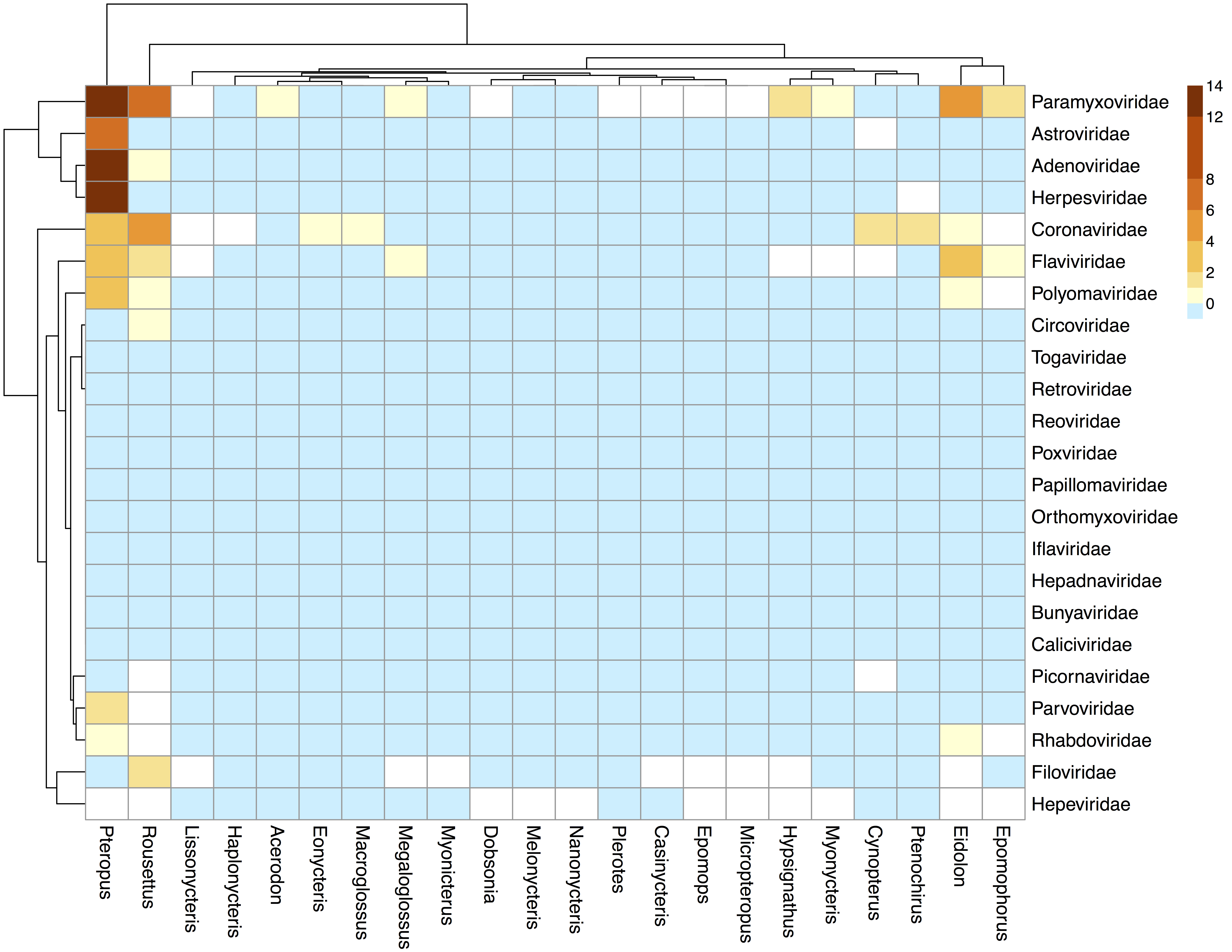

Supplement: S3 Fig — (DOCX) [file pone.0149237.s005.docx]
