# Supplementary material for: Optimizing Viral Discovery in Bats
Source: PLoS One. 2016 Feb 11;11(2):e0149237. doi: 10.1371/journal.pone.0149237 (PMC4750870; doi:10.1371/journal.pone.0149237)
Supplement: S1 Table — (DOCX) [file pone.0149237.s006.docx]

**Table S1. Databases/Reviews and filters used to find studies**

| **Database/Review** | **Search string and number of results** |
| --- | --- |
| PubMed | Filter by publication date: January 2007-June 2013  “bat AND virus”  “bat AND virus*”  “bat AND (virus OR viral)”  “bat AND (virus OR viral OR *virus) AND (novel OR emerging OR new) |
| Web of Science | Filter by year: 2007-2013  “bat AND virus”  Refined by: document type - article  “bat AND virus”  “bat AND (virus OR viral OR *virus) AND (novel OR emerging OR new) |
| Luis *et al*. 2013 review | 2007-current |
| Olival *et al.* 2012 | 2007-current |
