# Supplementary material for: Optimizing Viral Discovery in Bats
Source: PLoS One. 2016 Feb 11;11(2):e0149237. doi: 10.1371/journal.pone.0149237 (PMC4750870; doi:10.1371/journal.pone.0149237)
Supplement: S2 Table — (DOCX) [file pone.0149237.s007.docx]

**Table S2. Studies examined and study-level characteristics**

| **First Author** | **Year** | **Years Sampled** | **Source** | **# of Species** | **# of Bats** | **# of Bats Killed** | **# of Samples** | **# of Novel Viruses** | **# of Total Viruses** | **# of Viral Families** | **Reference #** |
| --- | --- | --- | --- | --- | --- | --- | --- | --- | --- | --- | --- |
| Aguilar-Setien, A | 2008 | 2005-2006 | wild-caught | 19 | 180 | 149 | 162 | 0 | 1 | 1 | (1) |
| Amengual, B | 2007 | 1995-2006 | wild-caught; already dead | 1 | 660 | 0 | 1069 | 0 | 1 | 1 | (2) |
| Anthony, S | 2013 | 2006-2010 | wild-caught | 1 | NA | 0 | 1897 | 34 | 55 | 9 | (3) |
| Anthony, S | 2013 | 2010 | wild-caught | 42 | 606 | 0 | 1046 | 12 | 13 | 1 | (4) |
| Ar Gouih, M | 2011 | 2006-2008 | wild-caught | 25 | NA | 0 | 552 | 2 | 2 | 1 | (5) |
| August, T | 2012 | 2006-2009 | wild-caught | 7 | 112 | 0 | 112 | 1 | 1 | 1 | (6) |
| Aznar-Lopez, C | 2013 | 2004-2010 | wild-caught | 27 | 1488 | 0 | 1526 | 0 | 1 | 1 | (7) |
| Baker, K | 2012 | 2010 | wild-caught | 1 | NA | 0 | 72 | 0 | 1 | 1 | (8) |
| Baker, K | 2013 | 2007, 2010 | wild-caught | 1 | 229 | 0 | 229 | 2 | 2 | 1 | (9) |
| Balboni, A | 2011 | 2009 | wild-caught | 1 | 52 | 0 | 52 | 1 | 1 | 1 | (10) |
| Banyard, A | 2009 | 2007-2008 | passive surveillance | 1 | 2 | 0 | NA | 0 | 1 | 1 | (11) |
| Brandao, P | 2008 | 2005 | wild-caught | 1 | 7 | 7 | 7 | 0 | 1 | 1 | (12) |
| Breed, A | 2013 | 2005 | wild-caught | 10 | 497 | 0 | NA | 0 | 3 | 1 | (13) |
| Ceballos, N | 2013 | 2011 | passive surveillance | 1 | 1 | 0 | NA | 1 | 1 | 1 | (14) |
| Chu, D | 2008 | 2004-2006 | wild-caught | 9 | 262 | 0 | 496 | 1 | 1 | 1 | (15) |
| Cui, J | 2008 | 2004-2007 | wild-caught | 8 | 336 | 336 | 1046 | 0 | 1 | 1 | (16) |
| Dominguez, S | 2007 | 2006 | wild-caught | 7 | 57 | 0 | 79 | 6 | 6 | 1 | (17) |
| Drexler, J | 2009 | 2008 | wild-caught | 1 | 215 | 0 | 215 | 3 | 3 | 1 | (18) |
| Drexler, J | 2010 | 2008 | wild-caught | 19 | 499 | 0 | 499 | 0 | 7 | 1 | (19) |
| Drexler, J | 2011 | 2008-2010 | wild-caught | 1 | NA | 0 | 382 | 1 | 8 | 3 | (20) |
| Drexler, J | 2012 | 2002-2011 | wild-caught; already dead | 85 | 3869 | 0 | 3869 | 5 | 5 | 1 | (21) |
| Drexler, J | 2012 | 2008-2010 | wild-caught | 86 | 4954 | 22 | 4954 | 66 | 159 | 1 | (22) |
| Du, L | 2010 | NA | wild-caught | 1 | 150 | 150 | 30 | 1 | 1 | 1 | (23) |
| Dzikwi, A | 2010 | 2006 | wild-caught | 5 | 350 | 350 | 490 | 0 | 2 | 1 | (24) |
| Emerson, G | 2013 | 2009-2011 | passive surveillance | 1 | 6 | 0 | NA | 1 | 1 | 1 | (25) |
| Epstein, J | 2010 | 2007 | wild-caught | 1 | 98 | 0 | 294 | 1 | 1 | NA | (26) |
| Fagrouch, Z | 2012 | NA | wild-caught | 22 | 163 | 163 | 163 | 3 | 3 | 1 | (27) |
| Falcon, A | 2011 | 2004-2007 | wild-caught | 26 | 576 | 0 | 606 | 0 | 14 | 1 | (28) |
| Freuling, C | 2011 | 2009 | passive surveillance | 1 | 1 | 0 | NA | 1 | 1 | 1 | (29) |
| Freuling, C | 2012 | 2006 | passive surveillance | 1 | 1 | 0 | NA | 0 | 1 | 1 | (30) |
| Ge, X | 2011 | 2009-2010 | wild-caught | 10 | 199 | NA | 199 | 5 | 22 | 1 | (31) |
| Geldenhuys, M | 2013 | 2005-2013 | archival tissues | 30 | 113 | 0 | 113 | 3 | 3 | 1 | (32) |
| Gloza-Rausch, F | 2008 | 2007 | wild-caught | 7 | 315 | 0 | 315 | 4 | 4 | 1 | (33) |
| Hayman, D | 2008 | 2007 | wild-caught | 6 | 206 | 0 | 206 | 0 | 2 | 1 | (34) |
| Hayman, D | 2008 | 2007 | wild-caught | 2 | 245 | 0 | 245 | 0 | 1 | 1 | (35) |
| Hayman, D | 2010 | 2008-2009 | wild-caught | 2 | 265 | 0 | 265 | 0 | 2 | 1 | (36) |
| He, B | 2013 | 2008 | already dead; market | 6 | 853 | 0 | 853 | NA | NA | 24 | (37) |
| He, B | 2013 | 2008 | already dead; market | 6 | 853 | 0 | 853 | 3 | 3 | 1 | (38) |
| Homaira, N | 2010 | 2007 | wild-caught | 1 | 118 | 0 | 118 | 0 | 1 | 1 | (39) |
| Iehl'e, C | 2007 | 2003-2005 | wild-caught; passive surveillance/archival tissue | 3 | 427 | 0 | 427 | 0 | 3 | 1 | (40) |
| Jiang, Y | 2010 | 2005-2006 | wild-caught | 8 | 685 | NA | 685 | 0 | 1 | 1 | (41) |
| Kohl, C | 2012 | 2009 | already dead; passive surveillance | 14 | 120 | 0 | NA | 3 | 3 | 1 | (42) |
| Kurth, A | 2012 | 2009 | already dead; passive surveillance | 15 | 120 | 0 | NA | 3 | 3 | 1 | (43) |
| Kuzmin, I | 2008 | 2006-2007 | wild-caught | 28 | 1221 | 1221 | 1182 | 0 | 1 | 1 | (44) |
| Kuzmin, I | 2010 | 2009 | wild-caught | 22 | 616 | 616 | 616 | 1 | 1 | 1 | (45) |
| Kuzmin, I | 2010 | 2007 | wild-caught | 1 | 272 | 272 | NA | 0 | 1 | 1 | (46) |
| Kuzmin, I | 2011 | 2009-2010 | wild-caught | 17 | 769 | 769 | NA | 0 | 2 | 1 | (47) |
| Lau, S | 2010 | 2005-2006 | wild-caught | 11 | 489 | 489 | 961 | 3 | 3 | 1 | (48) |
| Lau, S | 2011 | 2005 and 2008 | wild-caught | 18 | 1108 | 1108 | 2205 | 3 | 3 | 1 | (49) |
| Lau, S | 2012 | 2005-2010 | wild-caught | 22 | 4796 | 4796 | 9443 | 1 | 1 | 1 | (50) |
| Lelli, D | 2013 | 2010-2011 | wild-caught; passive surveillance; already dead | 9 | 112 | 0 | 156 | 0 | 1 | 1 | (51) |
| Li, Y | 2008 | 2004-2007 | wild-caught | 23 | 687 | NA | 692 | 0 | 1 | 1 | (52) |
| Li, Y | 2010 | 2007-2008 | wild-caught | 19 | 370 | NA | 370 | 1 | 1 | 1 | (53) |
| Lima, F | 2013 | 2012 | wild-caught | 2 | NA | 0 | 150 | 0 | 1 | 1 | (54) |
| Liu, Y | 2013 | 2012 | wild-caught | 5 | 261 | 261 | 261 | 0 | 1 | 1 | (55) |
| Maeda, K | 2008 | NA | wild-caught | 1 | 1 | 1 | NA | 1 | 1 | 1 | (56) |
| Misra, V | 2009 | 2007-2008 | wild-caught; passive surveillance; already dead | 8 | 115 | 115 | 115 | 1 | 2 | 7 | (57) |
| Muhldorfer, K | 2011 | 2002-2009 | passive surveillance | 19 | 486 | 0 | 210 | 0 | 2 | 6 | (58) |
| Muller, M | 2007 | 1986-1999 | wild-caught | 26 | 705 | 705 | 705 | 0 | 1 | 1 | (59) |
| Osborne, C | 2011 | 2007-2009 | wild-caught | 17 | 1044 | 0 | 1044 | 0 | 1 | 1 | (60) |
| Pajamo, K | 2008 | 2008 | passive surveillance | 1 | 1 | 1 | 1 | 0 | 1 | 1 | (61) |
| Pfefferle, S | 2009 | 2008 | wild-caught; captive | 12 | 335 | 0 | 335 | 0 | 2 | 1 | (62) |
| Philbey, A | 2008 | 1995-2000 | passive surveillance | 4 | 373 | 373 | 1659 | 0 | 1 | 1 | (63) |
| Pourrut, X | 2009 | 2003-2008 | wild-caught | 9 | 2147 | 2147 | 4022 | 0 | 2 | 1 | (64) |
| Quan, P | 2013 | 2007-2011 | wild-caught | 58 | 1615 | 498 | 1673 | 22 | 83 | 1 | (65) |
| Raut, C | 2012 | NA | wild-caught | 1 | 153 | 153 | 153 | 1 | 1 | 3 | (66) |
| Reusken, C | 2010 | NA | wild-caught | 13 | 211 | 0 | 211 | 0 | 1 | 1 | (67) |
| Rihtaric, D | 2010 | 2008 | wild-caught | 7 | 106 | 0 | 106 | 0 | 1 | 1 | (68) |
| Salmón-Mulanovich, G | 2009 | 2007 | wild-caught | 10 | 195 | 195 | 360 | 0 | 1 | 1 | (69) |
| Sasaki, M | 2012 | 2010 | wild-caught | 4 | 110 | 110 | 220 | 5 | 5 | 1 | (70) |
| Sendow, I | 2010 | NA | wildlife market | 2 | 99 | NA | 99 | 0 | 1 | 1 | (71) |
| Shirato, K | 2012 | 2009-2010 | wild-caught | 1 | 90 | 45 | 60 | 0 | 1 | 1 | (72) |
| Sonntag, M | 2009 | NA | already dead; passive surveillance | 12 | 55 | 0 | NA | 1 | 1 | 1 | (73) |
| Sumibcay, L | 2012 | 1981-2011 | archival tissues | 13 | 213 | 0 | 213 | 1 | 1 | 1 | (74) |
| Swanepoel, R | 2007 | 1999 | wild-caught | 8 | 525 | 525 | 525 | 0 | 1 | 1 | (75) |
| Tao, Y | 2013 | 2006 and 2009 | wild-caught | 35 | 291 | 291 | 380 | 7 | 12 | 1 | (76) |
| Thalmann, C | 2010 | 2002 | passive surveillance | 1 | 1 | 0 | NA | 1 | 2 | 3 | (77) |
| Tong, S | 2009 | 2006 | wild-caught | 21 | 221 | 221 | 221 | 7 | 8 | 1 | (78) |
| Tong, S | 2012 | 2009-2010 | wild-caught | 21 | 316 | 316 | 316 | 0 | 1 | 1 | (79) |
| Towner, J | 2007 | NA | wild-caught | 10 | 1142 | 1142 | 1138 | 0 | 1 | 1 | (80) |
| Towner, J | 2009 | 2007-2008 | wild-caught | 2 | 1220 | 1220 | 1220 | 0 | 2 | 1 | (81) |
| Tse, H | 2012 | NA | wild-caught | 14 | 728 | NA | 728 | 1 | 1 | 1 | (82) |
| Tsuda, S | 2012 | 2009, 2010, and 2011 | wild-caught | 8 | 179 | 179 | 179 | 2 | 2 | 1 | (83) |
| Wacharapluesadee, S | 2010 | 2005-2007 | wild-caught | 1 | NA | 0 | 2696 | 0 | 1 | 1 | (84) |
| Wacharapluesadee, S | 2013 | 2006-2007 | wild-caught | 14 | NA | 0 | 104 | 0 | 1 | 2 | (85) |
| Watanabe, S | 2009 | 2008 | wild-caught | 7 | 46 | 46 | 46 | 1 | 1 | 1 | (86) |
| Watanabe, S | 2010 | 2008-2009 | wild-caught | 1 | 58 | 58 | NA | 1 | 1 | 1 | (87) |
| Watanabe, S | 2010 | 2008 | wild-caught | 6 | 52 | 52 | 52 | 2 | 2 | 1 | (88) |
| Watanabe, S | 2010 | NA | wild-caught | 2 | 86 | 86 | 62 | 0 | 1 | 1 | (89) |
| Wibbelt, G | 2007 | NA | wild-caught; already dead | 7 | 25 | 0 | NA | 8 | 8 | 1 | (90) |
| Wilkinson, D | 2012 | NA | wild-caught | 15 | 197 | 197 | 197 | 0 | 2 | 1 | (91) |
| Wu, Z | 2012 | 2010-2011 | wild-caught | 11 | 216 | 0 | 432 | 13 | 13 | 23 | (92) |
| Yuan, J | 2010 | 2006 | wild-caught | 1 | 24 | 0 | 24 | 0 | 1 | 1 | (93) |

**Table 1 References**

1. Aguilar-Setién A, Romero-Almaraz ML, Sánchez-Hernández C, Figueroa R, Juárez-Palma LP, García-Flores MM, et al. Dengue virus in Mexican bats. Epidemiology and infection. 2008;136:1678-83.

2. Amengual B, Bourhy H, López-Roig M, Serra-Cobo J. Temporal dynamics of European bat Lyssavirus type 1 and survival of Myotis myotis bats in natural colonies. PloS one. 2007;2:e566.

3. Anthony SJ, Epstein JH, Murray KA, Navarrete-Macias I, Zambrana-Torrelio CM, Solovyov A, et al. A strategy to estimate unknown viral diversity in mammals. mBio. 2013;4(5):e00598-13.

4. Anthony SJ, Ojeda-Flores R, Rico-Chávez O, Navarrete-Macias I, Zambrana-Torrelio CM, Rostal MK, et al. Coronaviruses in bats from Mexico. The Journal of general virology. 2013;94:1028-38.

5. Gouilh MA, Puechmaille SJ, Gonzalez J-P, Teeling E, Kittayapong P, Manuguerra J-C. SARS-Coronavirus ancestor's foot-prints in South-East Asian bat colonies and the refuge theory. Infection, genetics and evolution : journal of molecular epidemiology and evolutionary genetics in infectious diseases. 2011;11:1690-702.

6. August T, Mathews F, Nunn M. Alphacoronavirus detected in bats in the United Kingdom. Vector borne and zoonotic diseases (Larchmont, NY). 2012;12:530-3.

7. Aznar-Lopez C, Vazquez-Moron S, Marston DA, Juste J, Ibáñez C, Berciano JM, et al. Detection of rhabdovirus viral RNA in oropharyngeal swabs and ectoparasites of Spanish bats. The Journal of general virology. 2013;94:69-75.

8. Baker KS, Todd S, Marsh G, Fernandez-Loras A, Suu-Ire R, Wood JLN, et al. Co-circulation of diverse paramyxoviruses in an urban African fruit bat population. The Journal of general virology. 2012;93:850-6.

9. Baker KS, Todd S, Marsh GA, Crameri G, Barr J, Kamins AO, et al. Novel, potentially zoonotic paramyxoviruses from the African straw-colored fruit bat Eidolon helvum. Journal of virology. 2013;87:1348-58.

10. Balboni A, Palladini A, Bogliani G, Battilani M. Detection of a virus related to betacoronaviruses in Italian greater horseshoe bats. Epidemiology and infection. 2011;139:216-9.

11. Banyard AC, Johnson N, Voller K, Hicks D, Nunez A, Hartley M, et al. Repeated detection of European bat lyssavirus type 2 in dead bats found at a single roost site in the UK. Archives of virology. 2009;154:1847-50.

12. Brandão PE, Scheffer K, Villarreal LY, Achkar S, Oliveira RdN, Fahl WdO, et al. A coronavirus detected in the vampire bat Desmodus rotundus. The Brazilian journal of infectious diseases : an official publication of the Brazilian Society of Infectious Diseases. 2008;12:466-8.

13. Breed AC, Meers J, Sendow I, Bossart KN, Barr JA, Smith I, et al. The Distribution of Henipaviruses in Southeast Asia and Australasia: Is Wallace's Line a Barrier to Nipah Virus? PloS one. 2013;8(4):e61316.

14. Ceballos NA, Morón SV, Berciano JM, Nicolás O, López CA, Juste J, et al. Novel lyssavirus in bat, Spain. Emerging infectious diseases. 2013;19:793-5.

15. Chu DKW, Poon LLM, Guan Y, Peiris JSM. Novel astroviruses in insectivorous bats. Journal of virology. 2008;82:9107-14.

16. Cui J, Counor D, Shen D, Sun G, He H, Deubel V, et al. Detection of Japanese encephalitis virus antibodies in bats in Southern China. The American journal of tropical medicine and hygiene. 2008;78:1007-11.

17. Dominguez SR, O'Shea TJ, Oko LM, Holmes KV. Detection of group 1 coronaviruses in bats in North America. Emerging infectious diseases. 2007;13:1295-300.

18. Drexler JF, Corman VM, Gloza-Rausch F, Seebens A, Annan A, Ipsen A, et al. Henipavirus RNA in African bats. PloS one. 2009;4:e6367.

19. Drexler JF, Gloza-Rausch F, Glende J, Corman VM, Muth D, Goettsche M, et al. Genomic characterization of severe acute respiratory syndrome-related coronavirus in European bats and classification of coronaviruses based on partial RNA-dependent RNA polymerase gene sequences. Journal of virology. 2010;84:11336-49.

20. Drexler JF, Corman VM, Wegner T, Tateno AF, Zerbinati RM, Gloza-Rausch F, et al. Amplification of emerging viruses in a bat colony. Emerging infectious diseases. 2011;17:449-56.

21. Drexler JF, Seelen A, Corman VM, Tateno AF, Cottontail V, Zerbinati RM, et al. Bats worldwide carry hepatitis E virus-related viruses that form a putative novel genus within the family Hepeviridae. Journal of virology. 2012;86:9134-47.

22. Drexler JF, Corman VM, Muller M, Maganga G, Vallo P, Binger T, et al. Bats host major mammalian paramyxoviruses. Nature communications. 2012;3:796.

23. Du L, Lu Z, Fan Y, Meng K, Jiang Y, Zhu Y, et al. Xi River virus, a new bat reovirus isolated in southern China. Archives of virology. 2010;155(8):1295-9.

24. Dzikwi AA, Kuzmin II, Umoh JU, Kwaga JKP, Ahmad AA, Rupprecht CE. Evidence of Lagos bat virus circulation among Nigerian fruit bats. Journal of wildlife diseases. 2010;46:267-71.

25. Emerson GL, Nordhausen R, Garner MM, Huckabee JR, Johnson S, Wohrle RD, et al. Novel Poxvirus in Big Brown Bats, Northwestern United States - Vol. 19 No. 6 - June 2013 - Emerging Infectious Disease journal - CDC. Emerg Infect Dis. 2013;19:1002-4.

26. Epstein JH, Quan P-L, Briese T, Street C, Jabado O, Conlan S, et al. Identification of GBV-D, a novel GB-like flavivirus from old world frugivorous bats (Pteropus giganteus) in Bangladesh. PLoS pathogens. 2010;6:e1000972.

27. Fagrouch Z, Sarwari R, Lavergne A, Delaval M, de Thoisy B, Lacoste V, et al. Novel polyomaviruses in South American bats and their relationship to other members of the family Polyomaviridae. The Journal of general virology. 2012;93(Pt 12):2652-7.

28. Falcón A, Vázquez-Morón S, Casas I, Aznar C, Ruiz G, Pozo F, et al. Detection of alpha and betacoronaviruses in multiple Iberian bat species. Archives of virology. 2011;156:1883-90.

29. Freuling CM, Beer M, Conraths FJ, Finke S, Hoffmann B, Keller B, et al. Novel lyssavirus in Natterer's bat, Germany. Emerging infectious diseases. 2011;17:1519-22.

30. Freuling CM, Kliemt J, Schares S, Heidecke D, Driechciarz R, Schatz J, et al. Detection of European bat lyssavirus 2 (EBLV-2) in a Daubenton's bat (Myotis daubentonii) from Magdeburg, Germany. Berliner und Munchener tierarztliche Wochenschrift. 2012;125:255-8.

31. Ge X, Li J, Peng C, Wu L, Yang X, Wu Y, et al. Genetic diversity of novel circular ssDNA viruses in bats in China. The Journal of general virology. 2011;92(Pt 11):2646-53.

32. Geldenhuys M, Weyer J, Nel LH, Markotter W. Coronaviruses in South african bats. Vector borne and zoonotic diseases (Larchmont, NY). 2013;13:516-9.

33. Gloza-Rausch F, Ipsen A, Seebens A, Göttsche M, Panning M, Felix Drexler J, et al. Detection and prevalence patterns of group I coronaviruses in bats, northern Germany. Emerging infectious diseases. 2008;14:626-31.

34. Hayman DTS, Suu-Ire R, Breed AC, McEachern JA, Wang L, Wood JLN, et al. Evidence of henipavirus infection in West African fruit bats. PloS one. 2008;3:e2739.

35. Hayman DTS, Fooks AR, Horton D, Suu-Ire R, Breed AC, Cunningham AA, et al. Antibodies against Lagos bat virus in megachiroptera from West Africa. Emerging infectious diseases. 2008;14:926-8.

36. Hayman DTS, Emmerich P, Yu M, Wang L-F, Suu-Ire R, Fooks AR, et al. Long-term survival of an urban fruit bat seropositive for Ebola and Lagos bat viruses. PloS one. 2010;5:e11978.

37. He B, Li Z, Yang F, Zheng J, Feng Y, Guo H, et al. Virome profiling of bats from myanmar by metagenomic analysis of tissue samples reveals more novel Mammalian viruses. PloS one. 2013;8(4):e61950.

38. He B, Fan Q, Yang F, Hu T, Qiu W, Feng Y, et al. Hepatitis virus in long-fingered bats, myanmar. Emerg Infect Dis. 2013;19(4):638-40.

39. Homaira N, Rahman M, Hossain MJ, Epstein JH, Sultana R, Khan MSU, et al. Nipah virus outbreak with person-to-person transmission in a district of Bangladesh, 2007. Epidemiology and infection. 2010;138:1630-6.

40. Iehlé C, Razafitrimo G, Razainirina J, Andriaholinirina N, Goodman SM, Faure C, et al. Henipavirus and Tioman virus antibodies in pteropodid bats, Madagascar. Emerging infectious diseases. 2007;13:159-61.

41. Jiang Y, Wang L, Lu Z, Xuan H, Han X, Xia X, et al. Seroprevalence of rabies virus antibodies in bats from southern China. Vector borne and zoonotic diseases (Larchmont, NY). 2010;10:177-81.

42. Kohl C, Lesnik R, Brinkmann A, Ebinger A, Radonic A, Nitsche A, et al. Isolation and characterization of three mammalian orthoreoviruses from European bats. PloS one. 2012;7:e43106.

43. Kurth A, Kohl C, Brinkmann A, Ebinger A, Harper JA, Wang L-F, et al. Novel paramyxoviruses in free-ranging European bats. PloS one. 2012;7:e38688.

44. Kuzmin IV, Niezgoda M, Franka R, Agwanda B, Markotter W, Beagley JC, et al. Lagos bat virus in Kenya. Journal of clinical microbiology. 2008;46:1451-61.

45. Kuzmin IV, Mayer A, Niezgoda M, Markotter W, Agwanda B, Breiman RF, et al. Shimoni bat virus, a new representative of the Lyssavirus genus. Virus research. 2010;149:197-210.

46. Kuzmin IV, Niezgoda M, Franka R, Agwanda B, Markotter W, Breiman RF, et al. Marburg virus in fruit bat, Kenya. Emerging infectious diseases. 2010;16:352-4.

47. Kuzmin IV, Turmelle AS, Agwanda B, Markotter W, Niezgoda M, Breiman RF, et al. Commerson's leaf-nosed bat (Hipposideros commersoni) is the likely reservoir of Shimoni bat virus. Vector borne and zoonotic diseases (Larchmont, NY). 2011;11(11):1465-70.

48. Lau SKP, Woo PCY, Wong BHL, Wong AYP, Tsoi H-W, Wang M, et al. Identification and complete genome analysis of three novel paramyxoviruses, Tuhoko virus 1, 2 and 3, in fruit bats from China. Virology. 2010;404:106-16.

49. Lau SKP, Woo PCY, Lai KKY, Huang Y, Yip CCY, Shek C-T, et al. Complete genome analysis of three novel picornaviruses from diverse bat species. Journal of virology. 2011;85:8819-28.

50. Lau SKP, Li KSM, Tsang AKL, Shek C-T, Wang M, Choi GKY, et al. Recent transmission of a novel alphacoronavirus, bat coronavirus HKU10, from Leschenault's rousettes to pomona leaf-nosed bats: first evidence of interspecies transmission of coronavirus between bats of different suborders. Journal of virology. 2012;86:11906-18.

51. Lelli D, Moreno A, Lavazza A, Bresaola M, Canelli E, Boniotti M, et al. Identification of Mammalian orthoreovirus type 3 in Italian bats. Zoonoses and public health. 2013;60:84-92.

52. Li Y, Wang J, Hickey AC, Zhang Y, Li Y, Wu Y, et al. Antibodies to Nipah or Nipah-like viruses in bats, China. Emerging infectious diseases. 2008;14:1974-6.

53. Li Y, Ge X, Hon C-C, Zhang H, Zhou P, Zhang Y, et al. Prevalence and genetic diversity of adeno-associated viruses in bats from China. The Journal of general virology. 2010;91:2601-9.

54. Lima FEdS, Campos FS, Kunert Filho HC, Batista HBdCR, Carnielli Júnior P, Cibulski SP, et al. Detection of Alphacoronavirus in velvety free-tailed bats (Molossus molossus) and Brazilian free-tailed bats (Tadarida brasiliensis) from urban area of Southern Brazil. Virus genes. 2013.

55. Liu Y, Zhang S, Zhao J, Zhang F, Hu R. Isolation of Irkut virus from a Murina leucogaster bat in China. PLoS neglected tropical diseases. 2013;7(3):e2097.

56. Maeda K, Hondo E, Terakawa J, Kiso Y, Nakaichi N, Endoh D, et al. Isolation of novel adenovirus from fruit bat (Pteropus dasymallus yayeyamae). Emerging infectious diseases. 2008;14:347-9.

57. Misra V, Dumonceaux T, Dubois J, Willis C, Nadin-Davis S, Severini A, et al. Detection of polyoma and corona viruses in bats of Canada. The Journal of general virology. 2009;90(Pt 8):2015-22.

58. Mühldorfer K, Speck S, Kurth A, Lesnik R, Freuling C, Müller T, et al. Diseases and causes of death in European bats: dynamics in disease susceptibility and infection rates. PloS one. 2011;6:e29773.

59. Müller MA, Paweska JT, Leman PA, Drosten C, Grywna K, Kemp A, et al. Coronavirus antibodies in African bat species. Emerging infectious diseases. 2007;13:1367-70.

60. Osborne C, Cryan PM, O'Shea TJ, Oko LM, Ndaluka C, Calisher CH, et al. Alphacoronaviruses in New World bats: prevalence, persistence, phylogeny, and potential for interaction with humans. PloS one. 2011;6:e19156.

61. Pajamo K, Harkess G, Goddard T. Isolation of European bat lyssavirus type 2 (EBLV-2) in a Daubenton's bat in the UK with a minimum incubation period of 9 months. Rabies Bulletin Europe. 2008.

62. Pfefferle S, Oppong S, Drexler JF, Gloza-Rausch F, Ipsen A, Seebens A, et al. Distant relatives of severe acute respiratory syndrome coronavirus and close relatives of human coronavirus 229E in bats, Ghana. Emerging infectious diseases. 2009;15:1377-84.

63. Philbey AW, Kirkland PD, Ross AD, Field HE, Srivastava M, Davis RJ, et al. Infection with Menangle virus in flying foxes (Pteropus spp.) in Australia. Australian veterinary journal. 2008;86:449-54.

64. Pourrut X, Souris M, Towner JS, Rollin PE, Nichol ST, Gonzalez J-P, et al. Large serological survey showing cocirculation of Ebola and Marburg viruses in Gabonese bat populations, and a high seroprevalence of both viruses in Rousettus aegyptiacus. BMC infectious diseases. 2009;9:159.

65. Quan P-L, Firth C, Conte JM, Williams SH, Zambrana-Torrelio CM, Anthony SJ, et al. Bats are a major natural reservoir for hepaciviruses and pegiviruses. Proceedings of the National Academy of Sciences of the United States of America. 2013;110:8194-9.

66. Raut CG, Yadav PD, Towner JS, Amman BR, Erickson BR, Cannon DL, et al. Isolation of a novel adenovirus from Rousettus leschenaultii bats from India. Intervirology. 2012;55(6):488-90.

67. Reusken CBEM, Lina PHC, Pielaat A, de Vries A, Dam-Deisz C, Adema J, et al. Circulation of group 2 coronaviruses in a bat species common to urban areas in Western Europe. Vector borne and zoonotic diseases (Larchmont, NY). 2010;10:785-91.

68. Rihtaric D, Hostnik P, Steyer A, Grom J, Toplak I. Identification of SARS-like coronaviruses in horseshoe bats (Rhinolophus hipposideros) in Slovenia. Archives of virology. 2010;155(4):507-14.

69. Salmón-Mulanovich G, Vásquez A, Albújar C, Guevara C, Laguna-Torres VA, Salazar M, et al. Human rabies and rabies in vampire and nonvampire bat species, Southeastern Peru, 2007. Emerging infectious diseases. 2009;15:1308-10.

70. Sasaki M, Setiyono A, Handharyani E, Rahmadani I, Taha S, Adiani S, et al. Molecular detection of a novel paramyxovirus in fruit bats from Indonesia. Virology journal. 2012;9:240.

71. Sendow I, Field HE, Adjid A, Ratnawati A, Breed AC, Darminto, et al. Screening for Nipah virus infection in West Kalimantan province, Indonesia. Zoonoses and public health. 2010;57:499-503.

72. Shirato K, Maeda K, Tsuda S, Suzuki K, Watanabe S, Shimoda H, et al. Detection of bat coronaviruses from Miniopterus fuliginosus in Japan. Virus genes. 2012;44(1):40-4.

73. Sonntag M, Mühldorfer K, Speck S, Wibbelt G, Kurth A. New adenovirus in bats, Germany. Emerging infectious diseases. 2009;15:2052-5.

74. Sumibcay L, Kadjo B, Gu SH, Kang HJ, Lim BK, Cook JA, et al. Divergent lineage of a novel hantavirus in the banana pipistrelle (Neoromicia nanus) in Côte d'Ivoire. Virology journal. 2012;9:34.

75. Swanepoel R, Smit SB, Rollin PE, Formenty P, Leman PA, Kemp A, et al. Studies of reservoir hosts for Marburg virus. Emerging infectious diseases. 2007;13:1847-51.

76. Tao Y, Shi M, Conrardy C, Kuzmin IV, Recuenco S, Agwanda B, et al. Discovery of diverse polyomaviruses in bats and the evolutionary history of the Polyomaviridae. The Journal of general virology. 2013;94(Pt 4):738-48.

77. Thalmann CM, Cummins DM, Yu M, Lunt R, Pritchard LI, Hansson E, et al. Broome virus, a new fusogenic Orthoreovirus species isolated from an Australian fruit bat. Virology. 2010;402:26-40.

78. Tong S, Conrardy C, Ruone S, Kuzmin IV, Guo X, Tao Y, et al. Detection of novel SARS-like and other coronaviruses in bats from Kenya. Emerging infectious diseases. 2009;15:482-5.

79. Tong S, Li Y, Rivailler P, Conrardy C, Castillo DAA, Chen L-M, et al. A distinct lineage of influenza A virus from bats. Proceedings of the National Academy of Sciences of the United States of America. 2012;109:4269-74.

80. Towner JS, Pourrut X, Albariño CG, Nkogue CN, Bird BH, Grard G, et al. Marburg Virus Infection Detected in a Common African Bat. PloS one. 2007;2:e764.

81. Towner JS, Amman BR, Sealy TK, Carroll SAR, Comer JA, Kemp A, et al. Isolation of genetically diverse Marburg viruses from Egyptian fruit bats. PLoS pathogens. 2009;5:e1000536.

82. Tse H, Chan WM, Li KS, Lau SK, Woo PC, Yuen KY. Discovery and genomic characterization of a novel bat sapovirus with unusual genomic features and phylogenetic position. PloS one. 2012;7(4):e34987.

83. Tsuda S, Watanabe S, Masangkay JS, Mizutani T, Alviola P, Ueda N, et al. Genomic and serological detection of bat coronavirus from bats in the Philippines. Archives of virology. 2012;157(12):2349-55.

84. Wacharapluesadee S, Boongird K, Wanghongsa S, Ratanasetyuth N, Supavonwong P, Saengsen D, et al. A longitudinal study of the prevalence of Nipah virus in Pteropus lylei bats in Thailand: evidence for seasonal preference in disease transmission. Vector borne and zoonotic diseases (Larchmont, NY). 2010;10:183-90.

85. Wacharapluesadee S, Sintunawa C, Kaewpom T, Khongnomnan K, Olival KJ, Epstein JH, et al. Group C betacoronavirus in bat guano fertilizer, Thailand. Emerging infectious diseases. 2013;19.

86. Watanabe S, Ueda N, Iha K, Masangkay JS, Fujii H, Alviola P, et al. Detection of a new bat gammaherpesvirus in the Philippines. Virus genes. 2009;39(1):90-3.

87. Watanabe S, Maeda K, Suzuki K, Ueda N, Iha K, Taniguchi S, et al. Novel betaherpesvirus in bats. Emerg Infect Dis. 2010;16(6):986-8.

88. Watanabe S, Masangkay JS, Nagata N, Morikawa S, Mizutani T, Fukushi S, et al. Bat coronaviruses and experimental infection of bats, the Philippines. Emerging infectious diseases. 2010;16:1217-23.

89. Watanabe S, Omatsu T, Miranda MEG, Masangkay JS, Ueda N, Endo M, et al. Epizootology and experimental infection of Yokose virus in bats. Comparative immunology, microbiology and infectious diseases. 2010;33:25-36.

90. Wibbelt G, Kurth A, Yasmum N, Bannert M, Nagel S, Nitsche A, et al. Discovery of herpesviruses in bats. The Journal of general virology. 2007;88:2651-5.

91. Wilkinson DA, Temmam S, Lebarbenchon C, Lagadec E, Chotte J, Guillebaud J, et al. Identification of novel paramyxoviruses in insectivorous bats of the Southwest Indian Ocean. Virus research. 2012;170:159-63.

92. Wu Z, Ren X, Yang L, Hu Y, Yang J, He G, et al. Virome analysis for identification of novel mammalian viruses in bat species from Chinese provinces. Journal of virology. 2012;86(20):10999-1012.

93. Yuan J, Hon C-C, Li Y, Wang D, Xu G, Zhang H, et al. Intraspecies diversity of SARS-like coronaviruses in Rhinolophus sinicus and its implications for the origin of SARS coronaviruses in humans. The Journal of general virology. 2010;91:1058-62.
