# Supplementary material for: Optimizing Viral Discovery in Bats
Source: PLoS One. 2016 Feb 11;11(2):e0149237. doi: 10.1371/journal.pone.0149237 (PMC4750870; doi:10.1371/journal.pone.0149237)
Supplement: S3 Table — (DOCX) [file pone.0149237.s008.docx]

**Table S3. Fitted generalized linear mixed models for data subsetted by viral family and detection method, with coefficients**

| **Subset of data** | **Model variables** | **Estimate (95% CI)** | **P value** |
| --- | --- | --- | --- |
| ***Adenoviridae*** |  |  |  |
| Molecular only (n=67) | SampleCatOther | 6.34x10^-4^ (0.00, Inf) | 0.9997 |
|  | SampleCatSaliva | 7.99 x10^7^ (0.00, Inf) | 0.9946 |
|  | SampleCatTissue | 2.17 x10^46^ (0.00, Inf) | 0.9932 |
|  | SampleCatUrine | 1.64 x10^8^ (0.00, Inf) | 0.9944 |
|  | ViralFamiliesTest | 0.0026 (0.00, Inf) | 0.9933 |
|  | N_Sample_Tested | 1.0025 (1.00046, 1.0045) | **0.0162** |
| ***Astroviridae*** |  |  |  |
| Molecular only (n=57) | SampleCatOther | 3.61 x10^6^ (0.00, Inf) | 0.9938 |
|  | SampleCatSaliva | 0.48 (0.039, 5.98) | 0.5722 |
|  | SampleCatTissue | 0.13 (0.0068, 2.40) | 0.1696 |
|  | SampleCatUrine | 6.16 (0.27, 139.27) | 0.2533 |
|  | HostFamilyMegadermatidae | 1.99 x10^-7^ (0.00, Inf) | 0.9969 |
|  | HostFamilyPteropodidae | 0.23 (0.0076, 6.70) | 0.3902 |
|  | HostFamilyRhinolophidae | 6.10 (0.17, 219.76) | 0.3225 |
|  | HostFamilyVespertilionidae | 18.38 (1.037, 325.94) | **0.0472** |
| ***Coronaviridae*** |  |  |  |
| Serology only (n=42) | N_Sample_Tested | 1.05 (0.99, 1.10) | 0.0774 |
| Molecular only (n=587) | SampleCatFeces | 17.66 (2.19, 142.50) | **0.0070** |
|  | SampleCatOther | 3.78 (0.20, 72.34) | 0.3769 |
|  | SampleCatSaliva | 4.11 (0.47, 36.20) | 0.2035 |
|  | SampleCatTissue | 1.57 (0.16, 15.54) | 0.6998 |
|  | SampleCatUrine | 2.30 (0.17, 30.41) | 0.5277 |
|  | SacrificedNum | 5.70 (2.69, 12.08) | **<0.0001** |
|  | HostFamilyEmballonuridae | 1.35 x10^5^ (0.00, Inf) | 0.9902 |
|  | HostFamilyHipposideridae | 1.54 x10^6^ (0.00, Inf) | 0.9881 |
|  | HostFamilyMegadermatidae | 3.39 x10^5^ (0.00, Inf) | 0.9894 |
|  | HostFamilyMolossidae | 1.20 x10^6^ (0.00, Inf) | 0.9883 |
|  | HostFamilyMormoopidae | 7.81 x10^5^ (0.00, Inf) | 0.9887 |
|  | HostFamilyNycteridae | 1.15 (0.00, Inf) | 0.9999 |
|  | HostFamilyPhyllostomidae | 2.02 x10^6^ (0.00, Inf) | 0.9879 |
|  | HostFamilyPteropodidae | 1.52 x10^6^ (0.00, Inf) | 0.9881 |
|  | HostFamilyRhinolophidae | 5.92 x10^5^ (0.00, Inf) | 0.9889 |
|  | HostFamilyVespertilionidae | 5.96 x10^5^ (0.00, Inf) | 0.9889 |
|  | ViralFamiliesTest | 1.27 (1.10, 1.47) | **0.0011** |
|  | N_Sample_Tested | 1.01 (1.00050, 1.0033) | **0.0079** |
| ***Flaviviridae*** |  |  |  |
| Serology only (n=24) | N_Sample_Tested | 1.08 (0.99, 1.17) | 0.0724 |
| Molecular only (n=149) | SampleCatFeces | 1.87 (0.11, 31.25) | 0.6614 |
|  | SampleCatOther | 1.33 x10^34^ (0.00, Inf) | 0.9927 |
|  | SampleCatSaliva | 1.62 x10^-8^ (0.00, Inf) | 0.9946 |
|  | SampleCatTissue | 0.15 (0.043, 0.50) | **0.0022** |
|  | ViralFamiliesTest | 0.067 (0.00, Inf) | 0.9916 |
|  | N_Sample_Tested | 1.01 (1.00049, 1.026) | **0.0419** |
| ***Hepeviridae*** |  |  |  |
| Molecular only (n=118) | Null (Intercept Only) | 0.044 (0.018, 0.11) | **<0.0001** |
| ***Herpesviridae*** |  |  |  |
| Molecular only (n=105) | SacrificedNum | 2.86 x10^-18^ (0.00, Inf) | 0.995 |
|  | HostFamilyPteropodidae | 1.24 x10^-28^ (0.00, Inf) | 0.997 |
|  | HostFamilyRhinolophidae | 1.98 x10^-18^ (0.00, Inf) | 0.998 |
|  | HostFamilyVespertilionidae | 2.56 x10^-18^ (0.00, Inf) | 0.998 |
|  | N_Sample_Tested | 1.0059 (1.0031, 1.0086) | **<0.0001** |
| ***Paramyxoviridae*** |  |  |  |
| Serology only (n=72) | SacrificedNum | 3.83 (0.69, 21.14) | 0.1231 |
|  | ViralFamiliesTest | 3.28 x10^-5^ (0.00, Inf) | 0.9887 |
|  | N_Sample_Tested | 1.031 (1.0076, 1.054) | **0.0088** |
| Molecular only (n=132) | SampleCatFeces | 9.25 x10^6^ (0.00, Inf) | 0.9912 |
|  | SampleCatSaliva | 1.88 (0.28, 12.90) | 0.5185 |
|  | SampleCatTissue | 6.74 (1.15, 39.65) | **0.0347** |
|  | SampleCatUrine | 1.37 (0.19, 9.76) | 0.7559 |
|  | ViralFamiliesTest | 0.69 (0.51, 0.92) | **0.0119** |
|  | N_Sample_Tested | 1.010 (1.0050, 1.015) | **0.0001** |
| ***Polyomaviridae*** |  |  |  |
| Molecular only (n=103) | SampleCatOther | 0.22 (0.023, 2.22) | 0.2011 |
|  | SampleCatSaliva | 1.28 x10^-11^ (8.48 x10^-20^, 0.0019) | **0.0090** |
|  | SampleCatTissue | 0.92 (0.26, 3.21) | 0.8965 |
|  | SampleCatUrine | 7.61 x10^-14^ (1.68 x10^-23^, 0.00035) | **0.0078** |
|  | ViralFamiliesTest | 0.74 (0.53, 1.028) | 0.0729 |
|  | N_Sample_Tested | 1.06 (1.02, 1.10) | **0.0037** |
| ***Rhabdoviridae*** |  |  |  |
| Serology only (n=97) | HostFamilyHipposideridae | 9.70 x10^7^ (0.00, Inf) | 0.9969 |
|  | HostFamilyMegadermatidae | 1.091 (0.00, Inf) | 1.0000 |
|  | HostFamilyMiniopteridae | 0.022 (0.00, Inf) | 0.9997 |
|  | HostFamilyMolossidae | 0.43 (0.00, Inf) | 0.9999 |
|  | HostFamilyNycteridae | 1.16 (0.00, Inf) | 1.0000 |
|  | HostFamilyPhyllostomidae | 5.48 x10^16^ (0.00, Inf) | 0.9959 |
|  | HostFamilyPteropodidae | 1.069 x10^8^ (0.00, Inf) | 0.9969 |
|  | HostFamilyRhinolophidae | 0.89 (0.00, Inf) | 1.0000 |
|  | HostFamilyVespertilionidae | 1.94 x10^7^ (0.00, Inf) | 0.9972 |
|  | N_Sample_Tested | 1.024 (1.0093, 1.038) | **0.0012** |
| Molecular only (n=71) | SampleCatSaliva | 2.56 x10^-7^ (0.00, Inf) | 0.9950 |
|  | SampleCatTissue | 1.29 x10^-5^ (0.00, Inf) | 0.9963 |
|  | SacrificedNum | 0.24 (0.034, 1.67) | 0.1478 |
|  | ViralFamiliesTest | 0.44 (0.17, 1.10) | 0.0788 |
|  | N_Sample_Tested | 1.01 (0.99, 1.016) | 0.0799 |
